# Supplementary material for: A herbicide resistance risk assessment for weeds in wheat and barley crops in New Zealand
Source: PLoS One. 2020 Jun 25;15(6):e0234771. doi: 10.1371/journal.pone.0234771 (PMC7316288; doi:10.1371/journal.pone.0234771)
Supplement: S1 Table — The list is derived from Bourdôt et al. [22], with additions from literature [23,24], expert knowledge and field observations made in January (late summer) of 2019 and 2020. Common name (in New Zealand) and family name are indicated. Nomenclature follows the New Zealand Flora [25]. </SI_Caption (DOCX) [file pone.0234771.s001.docx]

**Table S1.** **The full list of weed species considered in our risk assessment for herbicide resistance in New Zealand Wheat and Barley crops.** The species list is derived from Bourdôt et al. [1], with additions from literature [2,3], expert knowledge and field observations made in January (late summer) of 2019 and 2020. Common name (in New Zealand) and family name are indicated. Nomenclature follows the New Zealand Flora [4].

| **Species** | **Common Name** | **Family** |
| --- | --- | --- |
| 1. *Achillea millefolium* L. | yarrow | Asteraceae |
| 1. *Agrostis capillaris* L. | browntop | Poaceae |
| 1. *Amaranthus deflexus* L. | prostrate amaranth | Amaranthaceae |
| 1. *Amaranthus powellii* S.Watson | redroot, Powell's amaranth | Amaranthaceae |
| 1. *Amsinckia calycina* (Moris) Chater | yellow burweed | Boraginaceae |
| 1. *Aphanes arvensis* L. | parsley piert | Rosaceae |
| 1. *Arrhenatherum elatius* (L.) P.Beauv. ex J.Presl & C.Presl. | tall oat grass, onion twitch | Poaceae |
| 1. *Avena barbata* Link | slender wild oat | Poaceae |
| 1. *Avena fatua* L. | wild oat | Poaceae |
| 1. *Avena sterilis* L. | sterile oat, winter wild oat | Poaceae |
| 1. *Barbarea intermedia* Boreau | winter cress | Brassicaceae |
| 1. *Brassica napus* L. | rape | Brassicaceae |
| 1. *Brassica rapa* L. | wild mustard | Brassicaceae |
| 1. *Bromus catharticus* Vahl | prairie grass | Poaceae |
| 1. *Bromus diandrus* Roth | ripgut brome | Poaceae |
| 1. *Bromus hordeaceus* L. | soft brome | Poaceae |
| 1. *Bromus secalinus* L. | rye brome | Poaceae |
| 1. *Bromus sterilis* L. | poverty brome | Poaceae |
| 1. *Calandrinia compressa* Schrad. ex DC. | Curnow’s curse | Montiaceae |
| 1. *Calandrinia menziesii* (Hook.) Torr. & A.Gray | Curnow’s curse | Montiaceae |
| 1. *Capsella bursa-pastoris* (L.) Medik. | Sheppard’s purse | Brassicaceae |
| 1. *Cardamine flexuosa* With. | wavy bitter cress | Brassicaceae |
| 1. *Carduus nutans* L. | nodding thistle | Asteraceae |
| 1. *Cerastium glomeratum* Thuill. | annual mouse-ear chickweed | Caryophyllaceae |
| 1. *Chenopodiastrum murale* (L.) S.Fuentes, Uotila & Borsch | nettle-leaved fathen | Amaranthaceae |
| 1. *Chenopodium album* L. | fathen | Amaranthaceae |
| 1. *Cirsium arvense* (L.) Scop. | California thistle | Asteraceae |
| 1. *Cirsium vulgare* (Savi) Ten. | scotch thistle | Asteraceae |
| 1. *Convolvulus arvensis* L. | field bindweed | Convolvulaceae |
| 1. *Crepis capillaris* (L.) Wallr. | hawksbeard | Asteraceae |
| 1. *Crepis setosa* Haller f. | bristly hawksbeard | Asteraceae |
| 1. *Critesion murinum* L. | barley grass | Poaceae |
| 1. *Dactylis glomerata* L. | cocksfoot | Poaceae |
| 1. *Digitaria sanguinalis* (L.) Scop. | summer grass | Poaceae |
| 1. *Echinochloa crus-galli* (L.) P.Beauv. | barnyard grass | Poaceae |
| 1. *Elytrigia repens* (L.) Nevski | twitch | Poaceae |
| 1. *Erigeron bonariensis* L. | wavy-leaved fleabane | Asteraceae |
| 1. *Erigeron sumatrensis* Retz. | broadleaf fleabane | Asteraceae |
| 1. *Erodium cicutarium* (L.) L'Hér. | storksbill | Geraniaceae |
| 1. *Erodium moschatum* (L.) L'Hér. | musky storksbill | Geraniaceae |
| 1. *Fallopia convolvulus* (L.) Á.Löve | cornbind | Polygonaceae |
| 1. *Festuca rubra* L. | red fescue | Poaceae |
| 1. *Fumaria bastardii* Boreau | bastard’s fumitory | Papaveraceae |
| 1. *Fumaria densiflora* DC. | dense-flowered fumitory | Papaveraceae |
| 1. *Fumaria muralis* W.D.J.Koch | scrambling fumitory | Papaveraceae |
| 1. *Fumaria officinalis* L. | fumitory | Papaveraceae |
| 1. *Galium aparine* L. | cleavers | Rubiaceae |
| 1. *Gamochaeta coarctata* (Willd.) Kerg. | purple cudweed | Asteraceae |
| 1. *Gamochaeta purpurea* (L.) Cabrera | purple cudweed | Asteraceae |
| 1. *Geranium molle* L. | dove’s foot | Geraniaceae |
| 1. *Lactuca serriola* L. | prickly lettuce | Asteraceae |
| 1. *Lamium amplexicaule* L. | henbit | Lamiaceae |
| 1. *Leontodon saxatilis* Lam. | hawkbit | Asteraceae |
| 1. *Lepidium didymum* L. | twin cress | Brassicaceae |
| 1. *Lolium multiflorum* Lam. | annual ryegrass | Poaceae |
| 1. *Lolium perenne* L. | perennial ryegrass | Poaceae |
| 1. *Lotus pedunculatus* Cav. | lotus | Fabaceae |
| 1. *Lysimachia arvensis* (L.) U.Manns & Anderb. | scarlet pimpernel | Primulaceae |
| 1. *Malva neglecta* Wallr. | dwarf mallow | Malvaceae |
| 1. *Malva parviflora* L. | marshmallow | Malvaceae |
| 1. *Matricaria discoidea* DC. | pineapple weed | Asteraceae |
| 1. *Oxalis debilis* Kunth | pink shamrock | Oxalidaceae |
| 1. *Oxalis latifolia* Kunth | fishtail oxalis | Oxalidaceae |
| 1. *Persicaria maculosa* Gray | willow weed | Polygonaceae |
| 1. *Phalaris aquatica* L. | phalaris | Poaceae |
| 1. *Phalaris canariensis* L. | canary grass | Poaceae |
| 1. *Phalaris minor* Retz. | lesser canary grass | Poaceae |
| 1. *Phalaris paradoxa* L. | gnawed canary grass | Poaceae |
| 1. *Plantago lanceolata* L. | narrow-leaf plantain | Plantaginaceae |
| 1. *Poa annua* L. | poa | Poaceae |
| 1. *Polygonum aviculare* L. | prostrate knotweed | Polygonaceae |
| 1. *Ranunculus repens* L. | creeping buttercup | Ranunculaceae |
| 1. *Raphanus raphanistrum* L. | wild radish | Brassicaceae |
| 1. *Rumex acetosella* L. | sheep’s sorrel | Polygonaceae |
| 1. *Rumex obtusifolius* L. | broadleaf dock | Polygonaceae |
| 1. *Senecio vulgaris* L. | groundsel | Asteraceae |
| 1. *Sherardia arvensis* L. | field madder | Rubiaceae |
| 1. *Silene gallica* L. | catchfly | Caryophyllaceae |
| 1. *Silene vulgaris* (Moench) Garcke | bladder campion | Caryophyllaceae |
| 1. *Sisymbrium officinale* (L.) Scop. | hedge mustard | Brassicaceae |
| 1. *Solanum americanum Mill.* | American nightshade | Solanaceae |
| 1. *Solanum nigrum* L. | black nightshade | Solanaceae |
| 1. *Solanum sarrachoides* Sendtn. | hairy nightshade | Solanaceae |
| 1. *Sonchus asper* (L.) Hill | prickly sow thistle | Asteraceae |
| 1. *Sonchus oleraceus* L. | sow thistle | Asteraceae |
| 1. *Spergula arvensis* L. | spurrey | Caryophyllaceae |
| 1. *Stachys arvensis* (L.) L. | staggerweed | Lamiaceae |
| 1. *Stellaria media* (L.) Vill. | chickweed | Caryophyllaceae |
| 1. *Taraxacum officinale* F.H. Wigg. | dandelion | Asteraceae |
| 1. *Trifolium pratense* L. | red clover | Fabaceae |
| 1. *Trifolium repens* L. | white clover | Fabaceae |
| 1. *Tripleurospermum inodorum* (L.) Sch.Bip. | scentless mayweed | Asteraceae |
| 1. *Urtica urens* L. | dwarf nettle | Urticaceae |
| 1. *Veronica arvensis* L. | field speedwell | Plantaginaceae |
| 1. *Veronica persica* Poir. | scrambling speedwell | Plantaginaceae |
| 1. *Vicia hirsuta* (L.) Gray | hairy vetch | Fabaceae |
| 1. *Vicia lathyroides* L. | spring vetch | Fabaceae |
| 1. *Vicia sativa* L. | vetch | Fabaceae |
| 1. *Viola arvensis* Murray | field pansy | Violaceae |
| 1. *Vulpia bromoides* (L.) Gray | hairgrass | Poaceae |
| 1. *Vulpia myuros* (L.) C.C.Gmel. | ratstail fescue | Poaceae |

**References**

1. Bourdôt GW, Hurrell GA, Saville DJ. Weed flora of cereal crops in Canterbury, New Zealand. N Z J Crop Hortic Sci. 1998;26: 233–247. doi:10.1080/01140671.1998.9514059

2. Rolston MP, Archie WJ, Reddy K, Dastgheib F. Grass weed control and herbicide tolerance in cereals. N Z Plant Prot. 2003;56: 220–226. doi:10.30843/nzpp.2003.56.6095

3. Kon KF, Follas GB, James DE. Seed dormancy and germination phenology of grass weeds and implications for their control in cereals. N Z Plant Prot. 2007;60: 174–182. doi:10.30843/nzpp.2007.60.4597

4. Breitwieser I, Brownsey PJ, Heenan PB, Nelson WA, Wilton AD. Flora of New Zealand Online. 2010 [cited 15 Feb 2020]. Available: http://www.nzflora.info/
