# Supplementary material for: A herbicide resistance risk assessment for weeds in wheat and barley crops in New Zealand
Source: PLoS One. 2020 Jun 25;15(6):e0234771. doi: 10.1371/journal.pone.0234771 (PMC7316288; doi:10.1371/journal.pone.0234771)
Supplement: Attachment — Submitted filename: PONE-D-20-09801_R1 AUl.pdf [file pone.0234771.s005.pdf]

PLOS ONE

A herbicide resistance risk assessment for weeds in wheat and barley crops in New Zealand

--Manuscript Draft--

**Manuscript Number:**	PONE-D-20-09801R1
**Article Type:**	Research Article
**Full Title:**	A herbicide resistance risk assessment for weeds in wheat and barley crops in New Zealand
**Short Title:**	A herbicide resistance risk assessment for weeds in wheat and barley crops
**Corresponding Author:**	Chris Evan Buddenhagen<br>AgResearch Ltd Ruakura Research Centre<br>Hamilton, NEW ZEALAND
**Keywords:**	weed; herbicide-resistance; wheat; barley; risk-assessment; New Zealand
**Abstract:**	We estimated the risk of selecting for herbicide resistance in 101 weed species known to occur in wheat and barley crops on farms in New Zealand. A protocol was used that accounts for both the risk that different herbicides will select for resistance and each weed’s propensity to develop herbicide resistance based on the number of cases worldwide. To provide context we documented current herbicide use patterns. Most weeds (55) were low-risk, 30 were medium-risk and 16 high-risk. The top ten scored weeds were Echinochloa crus-galli, Poa annua, Lolium multiflorum, Erigeron sumatrensis, Raphanus raphanistrum, Lolium perenne, Erigeron bonariensis, Avena fatua, Avena sterilis and Digitaria sanguinalis. Seven out of ten high-risk weeds were grasses. The most used herbicides were synthetic auxins, an enolpyruvylshikimate-phosphate synthase inhibitor, acetolactate synthase (ALS) inhibitors, carotenoid biosynthesis inhibitors, and long-chain fatty acid inhibitors. ALS-inhibitors were assessed as posing the greatest risk for more species than other mode-of-action. Despite pre-emergence herbicides being known to delay resistance, New Zealand farmers only applied flufenacet and terbuthlazine with high frequency. Based on our analysis, surveys for herbicide-resistant species should focus on the high-risk species we identified. Farmer extension efforts in New Zealand should address resistance evolution in cropping weeds.
**Order of Authors:**	Zachary Ngow
	Richard J. Chynoweth
	Matilda Gunnarsson
	Phil Rolston
	Chris Evan Buddenhagen
**Opposed Reviewers:**	
**Response to Reviewers:**	Reviewer #1:<br>Because frequency and abundance of weed species in the region have not been used in the scoring, the paper provides a picture of risks due to the current use of herbicides in the re-gion, which is informative for the farmer about the intrinsic risk of an abundant species in its own farm. However, although I agree with intrinsic high risk species as stated on line 280-285, it is pity that the overall risk for the region is not presented.<br>The regional risk is assessed! We use a published account of weeds, as well as ex-pert knowledge to identify problem weeds in wheat and barley. The risk assess-ment considers the propensity of those weeds to develop resistance. It uses the most important factors, the crop specific region wide weed flora and region wide data about herbicide use. The selection pressure comes from the herbicides used and so captures the most important dimensions of the regional risk.<br><br>L50-54 There is also an important survey on blackgrass in UK (see Hicks et al Nature Ecology & Evolution 2(3) 2018, DOI: 10.1038/s41559-018-0470-1). Probably other reports are availa-ble on surveys developed by national weed research associations in

Spain, France (Columa), Italy, etc., including companies, extension service and farmers, and published in local con-ferences.
That is another example of the "focus on one or two problematic species in a given crop" issue we highlight, and therefore does not need to be added.

L142 It can be assumed that weed biology is already included in the inherent species score, but abundance is a key parameter. Abundance includes effects of farming systems: if many individuals still remain in the field, then the risk of developing resistance is higher than if the population is small. A score modifier to account for variation of abundance (e.g. 1 for low density and 3 for more than a million plant/ha, or another scale) could be used. Another factor is the species frequency in the studied region, which doesn't seem to be taken into account (is the Production Wise platform collecting main troublesome weed species?).
While that is true, regional abundance and density data do not exist to be able to implement that. We acknowledge that distribution, abundance and phenology are important #(L272). ProductionWise does not gather any information on weeds. Be-cause we identified high-risk species and herbicides the data are useful as a region-al assessment. Farmers and experts can now interpret the observed weed abun-dance with an eye to the likely risk of resistance.

Fig 2 Rather the low and high use HRAC groups I expected to see also the number of cases in New Zealand. I'm not clear with the low versus high use HRAC groups.

We emphasize high use herbicides in our studied regions, because that selection pressure drives the evolution of resistance. We mention in the introduction that in New Zealand, there are only 12 instances of herbicide resistance in arable crops #(L41-43) and in the discussion delve further into New Zealand HR weeds (L238-244). The high and low usage herbicide groups come from ProductionWise data, which can be found on Table 2. The basis of deciding which groups are 'high use in New Zealand' is on L106-108. Cases of resistance have been indicated on Table 3.

Reviewer #2 Dr Stephen Moss:

Abstract: Generally fine but as likely to be most-read part I suggest a few minor changes. Do you need to say 'wheat and barley' or would 'cereal' suffice? Ditto in title.
We think wheat and barley is a more accurate statement for our case since the herbicide data focuses on that. Wheat and barley is more widely grown than oats, and depending on the reader, corn would also be consid-ered to be a cereal…

I suggest:

15-18'We estimated the risk of selecting for herbicide resistance in 100 weed species known to occur in wheat and barley crops on farms in New Zealand. A protocol was used that accounts for both the risk that different herbicides will select for resistance and each weed's propensity to develop herbicide resistance based on the number of cases worldwide.' Done
24'ALS-inhibitors were assessed as posing the greatest risk for more species than any other mode-of-action. Done
25I prefer 'Pre-emergence' but that may depend on journal style. Done
26'……. in this class commonly used by…..' Is this better than 'favoured'? Done
Somewhere I think it would be worth stressing in abstract that 7 out of the high-risk 10 are grass-weeds. If space is limiting, I think the last sentence of abstract could go or 'farmer extension efforts' incorporated into previous sentence. Done
Introduction: Good – acceptably concise and relevant. I suggest including some brief (one or two sentences) information on arable cropping in NZ and what proportion of that is accounted for by wheat and barley. Or at least to show that wheat and barley comprise a significant proportion of arable crops – you might even wish to mention that NZ currently holds world record for both barley and wheat yield/ha (I think that is correct). I would! Done
32Suggest: '……potential losses….' Losses of 23% don't actually commonly occur. Done
41Clarify 'cases'. To non-specialist reader it is vague and could mean number of fields or farms – although 'specialists' know why that term is appropriate. I would suggest stating number of weed species instead which is 13 according to Heap website country

Powered by Editorial Manager® and ProduXion Manager® from Aries Systems Corporation

info. That lists 19 ‘cases’ (29 April) although that dupli-cates some species. If it is 25 cases now (according to your reference), perhaps someone in NZ should provide Ian Heap with updated info. This is important if this paper is published. Likewise amend ‘12’ in line 42 if appropriate. Done re-worded to reflect species numbers in New Zealand, and documented instances of resistance, to distinguish from the usage of cases in the Heap database.
45‘haphazard’ is a bit unfair, although this is a valid point. I would dispute that my sampling and testing over 30 years has been ‘haphazard’!   Suggest ‘unsystemat-ic’ as a better word – I would agree with that.   End sentence after ‘globally’. Then ‘It reflects…..’ Done
50comma needed after [9-14]. These 6 refs are all valid, but do you need them all? They are valid, focus on systematic efforts to detect any and all weed re-sistant cases. Also, they could be hard to find for other researchers working on a similar project. Leaving as is.
53-54Good points – one issue is that if resistance is perceived to be rare then hard to justify cost. If very widespread then why bother to do a survey? Money could be better spent. Also lack of infrastructure and personnel to conduct surveys may be as important as cost. Also, do surveys have much long-term value? Is a 20-year old survey of much value now – perhaps only as a benchmark? Perhaps money better spent on more ‘durable’ studies? Not saying that you need to con-sider these aspects here – perhaps in discussion as risk assessment relevant to survey priorities. Good points thanks for the insightful commentary.
59Suggest: ‘…….and their prior record of resistance in cereal fields elsewhere in the world.’ We specifically looked at wheat and barley. It’s an important dis-tinction.
62-64This is fine and a good succinct sentence, but I wonder if it is worth emphasising more that risk assessment includes not only herbicide risk but also weed species risk for non-specialist readers, as this is not particularly intuitive?   So, could read: ‘They present a quantitative risk matrix using both herbicide-risk (some herbicides pose a higher risk than others) and species-risk (some weed species are more resistance-prone than others), with an optional score modifier de-signed to account for agronomic management practices that may reduce the risk.’ Done
67Change: ‘Individual field and farm scale risk is not assessed as this requires de-tailed information on past herbicide use, including timing etc…..’ (This covers what has been applied - surely most important factor?) We changed the para-graph to emphasize importance of our herbicide application data set: “. This risk assessment is on an industry-wide scale informed by anonymized herbicide application data from wheat and barley fields. Risks were not assessed at the scale of individual farms and fields, this requires detailed information about herbicide timing, mixtures and rotations, and their interactions with weed bi-ology, crop rotations and other cultural practices. All the high-risk weeds iden-tified here should be targeted in surveys designed to detect herbicide-resistant weeds.
Materials and methods: Good – makes it clear what was done and why slightly differ-ent approaches to published protocol were adopted
76Suggest: ‘Most grasses and some …..’ The wonders of Google mean that I can see, within 60 seconds, that two grass weed species were identified at species level in the paper cited….. Done
83Suggest: ‘….legacy herbicide mode of action (MoA) groups……’ Done
88-89Suggest: ‘….legacy HRAC MoA group [24], with risk scores of 1, 2 or 3 given for low, medium or high risk respectively.’ Done
96Suggest: ‘…..scored as ‘1’.’ (‘One’ is a bit ambiguous). Done
98Suggest: ‘Most recent….’ Done but, “The most recent…”
108-109This is slightly confusing: ‘The most used herbicides for each crop were charac-terized by weighting active ingredient amounts and the number of fields they were applied to.’ It almost implies total a.i. weight was used. Not sure this sen-tence is needed. Deleted the sentence.
110Why taxon? Species better? Changed to species.
112Suggest: ‘…..HRAC MoA group…...’   And I suggest elsewhere throughout paper. Done
113Suggest: ‘….herbicide type…. Done
114Suggest: ‘……the global number of resistance cases….’ Done
115Suggest: ‘To obtain the ‘high’, ‘medium’ and ‘low’ risk scores as used in the Moss protocol [17],…..’ Done
117-118This sentence is slightly confusing and maybe could be improved. Is this correct?   ‘We assessed overall species-risk as the sum of the herbicide-risk multiplied

by the “inherent” species-risk [17] combined for all relevant HRAC MoA herbicide groups, but only…….’ (I think ‘once’ is confusing). The example you give below is useful. Done
120Suggest: ’……weed species….’ Done
135Suggest: ‘……to determine species-risk scores based on the number of cases of resistance worldwide, but we think this……’ Done
137Suggest: ‘ ….45 ‘high’ and ‘medium’ resistance risk species, many more than Moss et al……….’ Done
139Suggest: ‘The Moss protocol also used score modifiers that take into account re-sistance management practices including the use of non-chemical control measures’ . Done
139-142Suggest: ‘We did not use the score modifiers since these vary from field to field and our objective was slightly different. We acknowledge that actual risk of re-sistance development is determined mostly by the frequency and type of herbi-cide applied (selection pressure) interacting with characteristics of weed biolo-gy, distribution and abundance [5].’ Done
Results: Generally good and concise. One key paragraph needs improvement to im-prove clarity
145Suggest: ‘An additional 31 species were added…..’ Done
147Suggest: ‘ ….resulting in a total of 100 weed species for consideration.’ Done
155Suggest: Suggest: ‘This resembles the table of Moss et al., [17] with…’ Done
Table 1Use of ‘taxa’ is odd. Why not ‘species’ which is what is used on Heap’s website? Taxa is a rather more general term and I cannot see any justification for use here. Would it also be useful to make the herbicide example the most common-ly used ai for the group in NZ? Or if this has been done, state in title. Done
171-172Clarify if glyphosate used in-crop (for desiccation) or pre-sowing or both. Pre-sumably both, but some comment about balance of use would be useful if only to stress that glyphosate use is very different to selective herbicides – non-specialists might assume use is primarily in GM crops, as in some other coun-tries. Either here or earlier in M&M. Done “Glyphosate (MoA group G) is most-ly used (>95%) used pre-sowing of the cereal crops, for termination of the pre-vious crop or pre-establishment weed control. It is very rarely used as crop pre-harvest desiccant.”

Suggest: ‘ …….in barley (18%) compared with wheat (12%); conversely, farm-ers………’ Done
174Again, ‘HRAC MoA categories’ Done
Table 2Suggest last 10 categories are simply summarised to save space and some statement added within table. e.g. ‘The following 10 HRAC MoA groups each ac-counted for less than 1% of herbicide applications, N, F4, K1, I, H, F3, Z, L, F2 & K2.’ Done
181I don’t really see the point of stating actual % for barley when you haven’t for wheat. Why not simply add % values to each of the bars in Fig 1. You could then say a bit about relative use of some individual herbicides in wheat and barley. Somewhere you should give some indication of relative area sown with wheat and barley – in introduction or M&M. We added the percent values to the graph and clarified a comment about relative use under Table 2. The areas sown for wheat and barley are now in the introduction.
183Suggest: ‘Flufenacet and terbuthylazine were the only herbicides used on a sig-nificant area pre-emergence (the latter can also be used post-emergence), both used less in barley crops.’
185Suggest: ‘Fig 1. The ten most commonly applied herbicides in New Zealand wheat and barley fields.’ Done
190Why taxon? Surely ‘species’ is better? Done
193-194Say eight grasses and eight broad-leafed weeds. Done
201Suggest: ‘….five or more unique mode-of-action groups each’ What does>20 mean? Clarify. Done

202-215This paragraph covers the key output of this paper but is confusing and really needs a thorough re-write. Expand if necessary, to clarify results. Fig 2 contains a lot of information. I did wonder how much the ‘cases’ adds to this but I can see that it is relevant. Some definition of ‘cases’ is needed, as mentioned earlier. This is tricky as not easy to explain in a few words, but if this is explained in the M&M then no need to explain again, except perhaps to say refer to M&M. Should be made clear in Figure that cases is worldwide, as might be interpreted by casual reader as in NZ. Done

Figure 2 clearly mentions global cases. Also added the following to the methods: “Cases are defined by the International Sur-vey of Herbicide Resistant Weeds as unique combinations of weed species and HRAC herbicide mode-of-action (species x site of action).”
212-215I would make the point that 7 out of 10 are grass-weeds. Grass-weeds are over-represented in global cases of resistance. Some comment on this in discussion maybe. Done
Discussion: The paper would benefit from a more concise discussion with improved, more logical structure. Content is OK but lacking in focus and a bit ram-bling. I suggest reducing it to about 60% max of current length by heavily reducing some paragraphs or omitting altogether. The focus should be on the lessons and implications from what you have presented in the paper. Some of Discussion, while valid, reduces the impact of the paper rather than enhances it.
226-235Better if this is later in Discussion. Done
236-261Reduce this paragraph very substantially. Some of this could be in introduction.
There appear to be some contradictions quoted: ‘77 cases in wheat and 30 cases in barley [4].’ ‘ A review of the New Zealand literature shows only two reports of resistant species in wheat and barley [7,8].’ ‘Cases’ is confusing as different, I think, to previous use of term. Done
262-287Suggest this starts the Discussion as seems more logical. Done
288-306Again lacks focus. Glyphosate comments relevant. But to make some sugges-tion but then say ‘Estimations of these other factors can be made, but are likely to be inaccurate.’ Is a case of ‘shooting yourself in the foot.’ Ditto ‘Using Beck-ie’s as our herbicide-risk ranking would produce different risk-scores.’ Omit. Several sentences deleted and reworded to avoid “shooting ourselves in the foot” but keeping the main ideas as a reasonable caveat around interpretation of our results.
Other paragraphs – consider what content really adds to paper and what can be omitted.

One aspect that seems missing, is some idea of the crop rotations used on NZ farms and the impact this might have. This focus in this paper is on wheat and barley but the frequency of growing these crops and consequent herbicide use will surely impact on the resistance risk? How commonly is arable cropping ro-tated with grass? Are all-arable farms common compared with mixed farms? This may explain why herbicide resistance is relatively uncommon in NZ. Also relevant to risk at individual field level which lends itself nicely to comments about where you should set priorities for resistance monitoring, management and farmer KT. No need to go into great detail, but is relevant. We have includ-ed some text about rotations.

Conclusions: Generally fine but suggest the following.
343-346Suggest: ‘A European protocol [17], designed primarily to assist in herbicide authorization procedures, was adapted to assess the risk of herbicide re-sistance evolving in 100 different weed species known to occur in New Zealand wheat and barley crops. More than half the weeds (55) we assessed were low-risk, 29 were medium-risk and 16 high-risk. The 10 species posing the highest resistance risk were: etc etc’ Done
349-351Final two sentences could usefully be a bit ‘punchier’. ’Suggest: ‘We are plan-ning extensive surveys in New Zealand to detect new cases of herbicide re-sistance. The risk assessment outlined in this paper will enable us to prioritise those weeds identified as posing a high resistance risk and, consequently, make better use of available resources. The risk assessment procedure as described in this paper has the potential to be a very useful tool for evaluating the risk of herbicide resistance in a wide range of different weed species in other coun-tries too’. Done

Reviewer #3 (YASEEN KHALIL):
The introduction and methodology of the experiments seem to be satisfactory. However, in the methods section, the authors need to explain how they did the statistical analyses and data presentation.
We do not carry out any statistical tests. All our data is descriptive, and are visual-ized using graphs . We added a sentence to the last line of the methods section about the graphs.

Powered by Editorial Manager® and ProduXion Manager® from Aries Systems Corporation

	SPECIFIC COMMENTS:<br>L20-22 Add the classifiers to the scientific names. Alternatively, you may add the classifiers to the scientific names in the Materials and methods section and after that no need for it to be mentioned.<br>We think the reviewer is referring to the specific binomial author authorities. We examined other abstracts in PLOS ONE and do not see the these being used for ab-stracts unless it is specifically about a taxonomic treatment.<br>L20-22 Add hyphen “herbicide-resistant” Done but line 27<br>L45 Add the “it reflects the varying” Done<br>L52 Add the classifier to the scientific name “Alopecurus myosuroides ”. Done and also for Avena fatua<br>L20-22 Add hyphen “farm-scale” We added this at line 68 though which was the first men-tion<br>L74-76 What about the other 25% of the wheat and barley production regions in New Zea-land. I would prefer to stick to the mentioned region and not extrapolate to the whole coun-try of New Zealand.<br>We intend the weed list to capture our best estimate of the weeds known to occur in wheat and barley fields in New Zealand. We added a new sentence to explain: We expanded the weed species list to include species known to occur in wheat and barley fields in the wider New Zealand context.<br>L78 It will be very useful to mention the regions of the studies conducted by the mentioned researcher’s literature [21, 22].<br>We disagree. We checked the regions studied in those articles and knowing them adds lit-tle insight. Also, only a few species were added from these.<br>L79 It is recommended to follow the journal guidelines in this regards instead (Species no-menclature). Taxonomic authorities added at first mention.<br>L91-92 What is the logic and the rationale behind this decision? Worldwide, Group A herbi-cides are the most vulnerable group in terms of weed resistance evolving<br>We do classify Group A herbicides as high risk. We include a bit more detail here and consider the issues in the discussion. It now reads: With group A having 48 cas-es and group G herbicides having 47 cases we chose to place the two groups in the same risk category, with a difference in the numbers of cases of just worldwide we believe they are indistinguishable from the data. The alternative is to use the same threshold as in the Moss protocol, but this would result in group A and G being me-dium risk, which fails to capture the high-risk status of group A herbicides.<br>L122 Add the classifier to the scientific name “Chenopodium album”. Done<br>L140 Add the “that the actual” Done<br>L142 Add comma “distribution, and abundance” Done<br>L147 Add of “total of 100 weeds” Done, but it is now 101 weeds.<br>L150 I would recommend adding the S1 Table to the text instead of having it as a supporting file. Done<br><br>L152 I would recommend adding the S2 Table to the text instead of having it as a supporting file. Done We think this one should stay where it is because of its length.<br>L179 Add the “ranked by the wheat” Done<br>L203 Add comma “modes-of-action, and” Done<br>L234 Add comma “Without this list, we” Done<br>L234 replace are with a “As a result” Done<br>L240 Add comma “cases in wheat, and” Done<br>L246 Add s “indicates elevated” Not done. “may indicate” is correct<br>L247 Add comma “surprising when” Not done. Comma is correct<br>L256 delete “Clearly” Done<br>L275, 281, 284 Add hyphens to “spring-sown cereals” Done, “on-field” Not done the acting on field experience” refers to experience gained in the field, “high-risk” Done<br>L289“because of the number” Not done. This makes sense “This was necessary in part be-cause the number of cases for different herbicide groups has increased since the Moss arti-cle was published.”<br>L290 have instead of has “groups have increased” Not done. This makes sense “This was necessary in part because the number of cases for different herbicide groups has increased since the Moss article was published.”<br>L296“because of worldwide” Not done, it makes sense the way it is.<br>L308 Surprisingly, there have Done<br>L324 Add commas “may, in fact, implicate” Done
**Additional Information:**	

Question	Response
**Financial Disclosure**<br><br>Enter a financial disclosure statement that describes the sources of funding for the work included in this submission. Review the submission guidelines for detailed requirements. View published research articles from *PLOS ONE* for specific examples.<br><br>This statement is required for submission and **will appear in the published article** if the submission is accepted. Please make sure it is accurate.<br><br>**Unfunded studies**<br>Enter: *The author(s) received no specific funding for this work.*<br><br>**Funded studies**<br>Enter a statement with the following details:<br>• Initials of the authors who received each award<br>• Grant numbers awarded to each author<br>• The full name of each funder<br>• URL of each funder website<br>• Did the sponsors or funders play any role in the study design, data collection and analysis, decision to publish, or preparation of the manuscript?<br>• **NO** - Include this sentence at the end of your statement: *The funders had no role in study design, data collection and analysis, decision to publish, or preparation of the manuscript.*<br>• **YES** - Specify the role(s) played.<br><br>* typeset	All the authors worked under the Ministry of Business, Innovation and Employment [grant number C10X1806] to AgResearch Ltd. https://www.mbie.govt.nz/science-and-technology/science-and-innovation/funding-information-and-opportunities/investment-funds/endeavour-fund/<br>The funders had no role in study design, data collection and analysis, decision to publish, or preparation of the manuscript.
**Competing Interests**<br><br>Use the instructions below to enter a competing interest statement for this submission. On behalf of all authors, disclose any competing interests that could be perceived to bias this work—acknowledging all financial support and any other relevant financial or non-financial competing interests.<br><br>This statement **will appear in the published article** if the submission is	The authors have declared that no competing interests exist.

accepted. Please make sure it is accurate. View published research articles from *PLOS ONE* for specific examples.<br><br>**NO authors have competing interests**<br><br>Enter: *The authors have declared that no competing interests exist.*<br><br>**Authors with competing interests**<br><br>Enter competing interest details beginning with this statement:<br><br>*I have read the journal's policy and the authors of this manuscript have the following competing interests: [insert competing interests here]*<br><br>* typeset	
**Ethics Statement**<br><br>Enter an ethics statement for this submission. This statement is required if the study involved:<br><br>• Human participants<br>• Human specimens or tissue<br>• Vertebrate animals or cephalopods<br>• Vertebrate embryos or tissues<br>• Field research<br><br>Write "N/A" if the submission does not require an ethics statement.<br><br>General guidance is provided below. Consult the submission guidelines for detailed instructions. **Make sure that all information entered here is included in the Methods section of the manuscript.**	N/A

**Format for specific study types**<br><br>**Human Subject Research (involving human participants and/or tissue)**<br>• Give the name of the institutional review board or ethics committee that approved the study<br>• Include the approval number and/or a statement indicating approval of this research<br>• Indicate the form of consent obtained (written/oral) or the reason that consent was not obtained (e.g. the data were analyzed anonymously)<br><br>**Animal Research (involving vertebrate animals, embryos or tissues)**<br>• Provide the name of the Institutional Animal Care and Use Committee (IACUC) or other relevant ethics board that reviewed the study protocol, and indicate whether they approved this research or granted a formal waiver of ethical approval<br>• Include an approval number if one was obtained<br>• If the study involved *non-human primates*, add *additional details* about animal welfare and steps taken to ameliorate suffering<br>• If anesthesia, euthanasia, or any kind of animal sacrifice is part of the study, include briefly which substances and/or methods were applied<br><br>**Field Research**<br><br>Include the following details if this study involves the collection of plant, animal, or other materials from a natural setting:<br>• Field permit number<br>• Name of the institution or relevant body that granted permission	
**Data Availability**<br><br>Authors are required to make all data underlying the findings described fully available, without restriction, and from the time of publication. PLOS allows rare exceptions to address legal and ethical concerns. See the PLOS Data Policy and FAQ for detailed information.	Yes - all data are fully available without restriction

A Data Availability Statement describing where the data can be found is required at submission. Your answers to this question constitute the Data Availability Statement and **will be published in the article**, if accepted.<br><br>**Important:** Stating ‘data available on request from the author’ is not sufficient. If your data are only available upon request, select ‘No’ for the first question and explain your exceptional situation in the text box.<br><br>Do the authors confirm that all data underlying the findings described in their manuscript are fully available without restriction?	
**Describe where the data may be found in full sentences. If you are copying our sample text, replace any instances of XXX with the appropriate details.**<br><br>• If the data are **held or will be held in a public repository**, include URLs, accession numbers or DOIs. If this information will only be available after acceptance, indicate this by ticking the box below. For example: *All XXX files are available from the XXX database (accession number(s) XXX, XXX.)*.<br>• If the data are all contained **within the manuscript and/or Supporting Information files**, enter the following: *All relevant data are within the manuscript and its Supporting Information files.*<br>• If neither of these applies but you are able to provide **details of access elsewhere**, with or without limitations, please do so. For example:<br><br>*Data cannot be shared publicly because of [XXX]. Data are available from the XXX Institutional Data Access / Ethics Committee (contact via XXX) for researchers who meet the criteria for access to confidential data.*<br><br>*The data underlying the results presented in the study are available from (include the name of the third party*	All relevant data are within the manuscript and its Supporting Information files.

and contact information or URL).<br>• This text is appropriate if the data are owned by a third party and authors do not have permission to share the data.<br><br>* typeset	
Additional data availability information:	

Powered by Editorial Manager® and ProduXion Manager® from Aries Systems Corporation

agresearch
āta mātai, mātai whetū

18 May 2020

Dear PLOS ONE editors (Ahmet Uludag, Ph.D.),

Thanks for shepherding our manuscript "A herbicide resistance risk assessment for weeds in wheat and barley crops in New Zealand" through the review process. We are happy to provide you with a much-improved manuscript. Reviewer 1 seemed to focus on specific concerns about aspects of the methods. After a careful re-read of the relevant parts of the article we think those concerns are addressed in the article. We found the comments of Reviewer 2 (Dr. Stephen Moss, whose protocol we adapted) were very helpful, and because of his suggestions we reorganised the discussion substantially and adopted most of his other recommendations. Reviewer 3 also provided helpful suggestions that were mostly adopted. We provide the following line-by-line responses to the reviewers. The most significant of these was to move both the supplemental data files into the main text. We did this for one of them but left the species list as a supplement. It's a big table, so we would defer to your judgement about including it. In the "Response_To_Reviewers.doc" document reviewer comments are followed by our response in ***bold italics***. We also added *Solanum americanum*, a species we should have included in our assessment bringing the number of species assessed up to 101 – relevant updates were made throughout the text.

We do not think the text needs to be changed with respect to ZN and CEB affiliations. If you think it needs to be changed it could include this information: AgResearch Ltd is a crown owned research institution doing science research businesses but owned by the Crown (i.e. the Government) in New Zealand. ZN and CEB affiliation to AgResearch Ltd. does not alter our adherence to PLOS ONE policies on sharing data and materials. The funding agency is also the main public science funding organization in New Zealand and provided financial support in the form of authors' salaries and/or research materials. They did not play a role in the study design, data collection and analysis, decision to publish, or preparation of the manuscript.

We have had one prior interaction with PLOS about this manuscript when we first submitted it – our previous cover letter was dated 6 April 2020.

Sincerely,

Chris Buddenhagen
chris.buddenhagen (skype)
+64 221 04084 (cell)
Chris.Buddenhagen@agresearch.co.nz

Manuscript Click here to access/download;Manuscript;Manuscript.docx

A herbicide resistance risk assessment for weeds in wheat and barley crops in New Zealand

Zachary Ngow[1*¶], Richard J. Chynoweth[2&], Matilda Gunnarsson[2&], Phil Rolston[2&], Christopher E. Buddenhagen[1*¶]

[1] AgResearch Ltd., Hamilton, New Zealand

[2] Foundation for Arable Research, Christchurch, New Zealand

* Corresponding Authors: E-mail: zacharyngow@gmail.com, E-mail: chris.buddenhagen@agresearch.co.nz

¶ These authors contributed equally to this work.

& These authors also contributed equally to this work.

Abstract

We estimated the risk of selecting for herbicide resistance in 101 weed species known to occur in wheat and barley crops on farms in New Zealand. A protocol was used that accounts for both the risk that different herbicides will select for resistance and each weed's propensity to develop herbicide resistance based on the number of cases worldwide. To provide context we documented current herbicide use patterns. Most weeds (55) were low-risk, 30 were medium-risk and 16 high-risk. The top ten scored weeds were *Echinochloa crus-galli, Poa annua, Lolium multiflorum, Erigeron sumatrensis, Raphanus raphanistrum, Lolium perenne, Erigeron bonariensis, Avena fatua, Avena sterilis* and *Digitaria sanguinalis*. Seven out of ten high-risk weeds were grasses. The most used herbicides were synthetic auxins, an enolpyruvylshikimate-phosphate synthase inhibitor, acetolactate synthase (ALS) inhibitors, carotenoid biosynthesis inhibitors, and long-chain fatty acid inhibitors. ALS-inhibitors were assessed as posing the greatest risk for more species than other mode-of-action. Despite pre-emergence herbicides being known to delay resistance, New Zealand farmers only applied flufenacet and terbuthlazine with high frequency. Based on our analysis, surveys for herbicide-resistant species should focus on the high-risk species we identified. Farmer extension efforts in New Zealand should address resistance evolution in cropping weeds.

Introduction

A worldwide analysis suggests that weeds have the highest potential to cause yield losses in major crops including wheat and barley where they account for potential losses of 23% [1,2]. In response to this threat to production, farmers have come to rely on a mix of cultural and chemical control methods. Herbicidal weed control is favoured as it is cost-effective providing a three or four-fold economic return [2] and is a key element in the implementation of conservation tillage systems (including direct drilling) which reduce soil erosion [3]. However, intensive herbicide use is known to select for resistant individuals in weed populations, eroding herbicide effectiveness as these weeds escape control [4–6]. Globally farmers are addressing similar suites of weed species in any given crop, and some appear to have a greater propensity to develop resistance, with repeated convergent evolution of resistance being documented in the International Survey of Herbicide Resistant Weeds [4] and the scientific literature generally [5,6]. Over the last 40 years a total of 14 species of herbicide resistant weeds have been documented in New Zealand, with 12 [illegible] resistance documented in arable crops including maize, peas, oats, wheat, and barley [7]. In any given year >50% of New Zealand arable production areas are under wheat (~45000 ha) and barley (~55000 ha) rotations [8]. Production levels are high, with farmers in New Zealand obtaining world record wheat and barley yields in 2017 and 2015, respectively [9,10].

An examination of weed science publications shows that our knowledge about herbicide resistance cases has developed mostly via unsystematic detection globally. It reflects the varying effort, scientific input, and methods of detection; definitely this is the case in New Zealand [7,11]. This makes sense since farmers and herbicide companies do not necessarily report resistance cases to scientists, and even if they do, not all cases are likely to get published. What's more, ad-hoc reporting may reflect strong biases towards a small number of the most problematic weeds. With some notable exceptions, particularly in cropping systems in Australia [12–17], systematic surveys to detect

herbicide resistance cases are rare, and often focus on one or two problematic species in a given crop, for example, *Alopecurus myosuroides* Huds. in French wheat fields [18] or *Avena fatua* L. in two Canadian townships [19]. Systematic surveys may be rare because of the cost, a lack of specific pathways for reporting [11], and industry perceptions about the importance of resistance. Surveyors should ideally be open to the discovery of completely novel cases while also being aware of those that are most at risk of developing resistance.

Here we adapt a recently published risk assessment protocol [20] to identify those weeds most at risk of developing resistance in New Zealand, given their occurrence in wheat and barley fields, and their prior record of resistance in wheat and barley fields elsewhere in the world. This Moss et al. protocol [20] set out to assess resistance risk as part of a pesticide authorization process in Europe, based on a European Plant Protection Organization (EPPO) protocol, originally developed in 1999 [21]. They present a quantitative risk matrix using both herbicide-risk (some herbicides pose a higher risk than others) and species-risk (some weed species are more resistance-prone than others), with an optional score modifier designed to account for agronomic management practices that may reduce the risk. We took advantage of a unique data set about herbicide use in wheat and barley fields in New Zealand to place our risk assessment into context, and construct a framework for herbicide resistance surveys and extension efforts in the New Zealand cropping industry. This risk assessment is on an industry-wide scale informed by anonymized herbicide application data from wheat and barley fields. Risks were not assessed at the scale of individual farms and fields, this requires detailed information about herbicide timing, mixtures and rotations, and their interactions with weed biology, crop rotations and other cultural practices. All the high-risk weeds identified here should be targeted in surveys designed to detect herbicide-resistant weeds.

Materials and Methods

Weed list

We generated a list of potential target weeds from wheat and barley crops in New Zealand. This was primarily sourced from the Bourdôt et al. 1998 weed survey in New Zealand (Canterbury region) wheat and barley fields [22]. The Canterbury and northern Otago regions contain more than 75% of all the wheat and barley grown in New Zealand [8]. We expanded the weed species list to include species known to occur in wheat and barley fields in the wider New Zealand context. Grasses and some broadleaf genera were not identified to species by Bourdôt et al. [22], hence we took steps to address this gap and other omissions by using other literature [23,24], expert knowledge and field observations made in January (late summer) of 2019 and 2020. Species nomenclature follows the New Zealand Flora and taxonomic authorities are listed in Table S1. [25]. Subspecies were not distinguished, and taxa were considered by us only at the species level (e.g. *Avena sterilis* subsp. *ludoviciana* (Durieu) M.Gillet & Magne was treated as *Avena sterilis L.*).

Ranking herbicide groups by resistance cases

We ranked Herbicide Resistance Action Committee (HRAC) legacy herbicide mode of action (MoA) groups by the number of resistance cases documented by the International Survey of Herbicide

Resistant Weeds [4] to obtain an estimate of what Moss et al. called the "inherent risk" of the herbicide [20]. The Moss et al. protocol [20] (henceforth the "Moss protocol") considers the inherent risk to relate to the total number of cases of resistance reported in the International Survey of Herbicide Resistant Weeds [4] for each legacy HRAC MoA [26], with risk scores of 1, 2 or 3 given for low, medium or high risk respectively. We set the threshold for high-risk at >9% of recorded cases, which captures the original high-risk categories identified in the Moss protocol, but now places group G (glyphosate) in the high category. Moss et al. used the 10% threshold for high-risk herbicides. With group A having 48 cases and group G herbicides having 47 cases we chose to place the two groups in the same risk category, with a difference in the numbers of cases of just worldwide we believe they are indistinguishable from the data. The alternative is to use the same threshold as in the Moss protocol, but this would result in group A and G being medium risk, which fails to capture the high-risk status of group A herbicides. There are different ways we can reasonably set risk thresholds, which are discussed later. Remaining ranking thresholds were not changed: medium risk 5-9%, low risk 1-5% and very low risk <1%. Low-risk and very low risk are both scored as '1'.

Herbicide use

The most recent (2017-2018) data on herbicide usage trends in New Zealand were sourced from the ProductionWise® [27] platform and aggregated by active ingredient. This consisted of data entered by farmers about herbicide use in 5026 barley and 7647 wheat fields. Approximately 900 arable farmers have registered to use the platform, but anonymization was complete, with no unique identifiers for farms or fields. Farmers recorded every spray event (by herbicide product) in their fields. For example, an individual active ingredient used three times in a field is recorded three times. Products with multiple active ingredients were recorded as independent applications. Counts of herbicide use in fields were summarized by active ingredient and legacy HRAC [26] herbicide mode-of-action, from product label information. Relative rates of use by mode-of-action were quantified and characterized as very high (>20% of all application instances), high (>10%), moderate (>1%), low (~1%), extremely low (<1%) and nonexistent (0%).

Cases of resistance by taxon and risk scoring

We assume that the best way to predict resistance in a weed species to any given herbicide (by HRAC group) is proportional to the number of documented cases of herbicide resistance in the same taxon, given the use of the same herbicide type in New Zealand. To calculate Moss's "inherent" species-risk scores we used the global number of resistance cases from the International Survey of Herbicide Resistant Weeds [4]. Cases are defined by the International Survey of Herbicide Resistant Weeds as unique combinations of weed species and HRAC herbicide mode-of-action (species x site of action). To obtain the 'high', 'medium' and 'low' risk scores as used in the Moss protocol [20], we designated ≥10 cases as high risk (score=3), < 10 as moderate (score=2) and no cases recorded as low risk (score=1). We assessed overall species-risk as the sum of the herbicide-risk multiplied by the "inherent" species-risk [20] combined for all relevant HRAC MoA herbicide groups, but only, but only where species had cases of resistance documented somewhere in the world. We include all cases of resistance for each weed species, rather than restricting our focus to cases from wheat and barley, because we are interested in a species propensity to develop

resistance to a herbicide group. For example, the high-risk species *Chenopodium album* L. has more than 10 documented cases of resistance giving it a species score of 3. Then we consider the herbicide-risk scores for those herbicides where *Chenopodium album* has evolved resistance somewhere in the world. There were cases in two high-risk herbicide groups B and C1 (each with a herbicide score of 3), and one medium-risk group O (herbicide score of 2). The species and herbicide scores are multiplied and summed (3×3) + (3×3) + (3×2) = 24. We distinguished cases that were in herbicide groups highly-used (or not) by wheat and barley farmers in New Zealand.

The summed (cumulative) scoring method described above is not used in the European Moss protocol because its purpose was to regulate herbicide product use [20]. In contrast, we wanted to determine the risk that different herbicides will select for resistance in weed species known to occur in New Zealand’s wheat and barley fields. Ultimately we hope to inform sector stakeholders about risk, and to improve herbicide resistance detection. We adapted their protocol to score species-risk in an industry-wide assessment. Unlike Moss et al. [20] we used an explicit threshold (though arbitrary) to determine species-risk scores based on the number of cases of resistance worldwide, but we think this approach produces credible risk estimates in the light of current knowledge. We examined 101 species and ended up with 46 ‘high’ and ‘medium’ resistance risk species, many more than Moss et al (they scored an example list of only 13 high and medium risk taxa). The Moss protocol also used score modifiers that take into account resistance management practices including the use of non-chemical control measures. We did not use the score modifiers since these vary from field to field and our objective was slightly different. We acknowledge that actual risk of resistance development is determined mostly by the frequency and type of herbicide applied (selection pressure) interacting with characteristics of weed biology, distribution and abundance [5]. All graphs were created in R using the ggplot2 package [28,29].

Results

Weed list

Our weed list contained 69 species from the original Bourdôt et al. article [22]. An additional 32 species were added based on field observation, expert opinion and relevant literature [23,24], resulting in a total of 101 weed species for consideration. *Digitaria sanguinalis*, *Echinochloa crus-galli, Erigeron* spp. and *Raphanus raphanistrum* are notable emerging weeds, so they were included. Some taxa noted by Bourdôt were resolved to species, such as *Trifolium* spp., which became *Trifolium repens* and *Trifolium pratense*. A full list of weeds considered for New Zealand wheat and barley crops is displayed in Table S1.

Ranking herbicide groups by resistance cases

Herbicide mode-of-action risk rankings are displayed (Table 1), arranged from high risk to very low risk. This resembles the table of Moss et al., [20] with a notable exception in that group G is raised to high risk. We designated HRAC groups B, C1, A, G as high-risk (>9% of recorded cases), O, D, C2 as moderate (5-9%), E, K1, K3, N F, Z as low (1-5%) and C3, F1, H, L as very low risk (<1%).

Table 1. Herbicide mode-of-action groups ranked by resistance risk. The number of cases of resistance from the International Survey of Herbicide Resistant Weeds [4] ranked and grouped by the legacy HRAC mode-of-action (data accessed in January 2020). [illegible]cludes subcategories Z1, Z2, Z3, Z4. Othe[illegible]ncludes mode-of-actions with 2 or fewer cases: F2, F4, K2, I. The example active ingredients are examples of commonly used active ingredients in the New Zealand context.

Resistance Risk	HRAC Herbicide MoA Groups		Example active ingredient	Number of resistant species worldwide	% of the worldwide total
High	B	ALS inhibitor	flumetsulam	165	32
	C1	PSII inhibitors (triazines)	atrazine	74	15
	A	ACCase inhibitors	pinoxaden	48	9
	G	EPSP synthase inhibitors	glyphosate	47	9
Medium	O	Synthetic auxin	MCPA	41	8
	D	PSI electron diverters	paraquat	32	6
	C2	PSII inhibitors (ureas and amides)	isoproturon	29	6
Low	E	PPO inhibitors	carfentrazone	13	3
	K1	Microtubule inhibitors	trifluralin	12	2
	N	Lipid inhibitors	triallate	10	2
	K3	Long-chain fatty acid inhibitors	flufenacet	7	1
	F3	Carotenoid biosynthesis (unknown target)	amitrole	6	1
	Z[illegible]	Anti-microtubule mitotic disrupter	flamprop	6	1
Very-low	C3	PSII inhibitors (nitriles)	ioxynil	4	<1
	F1	Carotenoid biosynthesis inhibitors	diflufenican	4	<1
	H	Glutamine synthase inhibitors	glufosinate	4	<1
	L	Cellulose inhibitors	dichlobenil	3	<1
	-	Other [illegible]	-	6	1

Herbicide use

Current herbicide use in wheat and barley fields [illegible]nvolves 75 unique active ingredients in 16 mode-of-action groups (we show the most importan[illegible]rbicide groups in Table 2). Barley and wheat have shared patterns of herbicide usage with respect to mode-of-action (Table 2) and active-ingredients. Synthetic auxins (HRAC group O) were represented in higher proportions than any other class of herbicide (a total usage rate of 26%). ALS-inhibitors (B), PDS-inhibitors (F1) and EPSPS-inhibitors (G) were highly used herbicide groups with >10% total usage, and acetyl coenzyme-A carboxylase inhibitors (A) and photosystem-II disrupters (C) were moderately used (total <10%).

EPSPS-inhibitors (G) were used in larger proportions in barley (18%) compared to wheat (12%); conversely, farmers used K3 herbicides significantly more in wheat at 12% compared to barley at 4%. Glyphosate (MoA group G) is mostly used (>95%) used to control weeds pre-sowing of the cereal crops, for termination of the previous crop or pre-establishment weed control. It is very rarely used as crop pre-harvest desiccant.

Table 2. **Ranked herbicide mode-of-action usage proportions.** The percentage of fields that received herbicide applications, grouped by HRAC MoA categories. Data are sourced from the ProductionWise® platform, entered by farmers. It represents a record of herbicide use from 5026 barley and 7647 wheat fields, collected during 2017 and 2018. The following 10 HRAC MoA groups each accounted for less than 1% of herbicide applications, N, F4, K1, I, H, F3, Z, L, F2 & K2.

Mode-of-action	Barley %	Wheat %	Total %
O	30	24	26
B	16	17	16
F1	14	17	16
G	18	12	14
K3	4	12	9
A	8	6	7
C1	3	5	4
C2	1	4	3
C3	2	1	1
E	2	<1	1
D	1	1	1

Individual herbicide usage ranking for each crop is displayed in Fig. 1, ranked by the wheat herbicide use. The ten most used herbicides (in order) for barley were: glyphosate (HRAC group G), diflufenican (F1), fluroxypyr (O), MCPA (O), chlorsulfuron (B), pinoxaden (A), iodosulfuron (B), flufenacet (K3), mecoprop (O) and clopyralid (O). Flufenacet and terbuthylazine were the only high-use pre-emergent active-ingredient used in wheat (the latter can also be used post-emergent), both were used less in barley crops.

Fi[illegible] The ten most common herbicides applied in New Zealand wheat and barley fields. The ten most used herbicides as a percentage of total application instances documented by farmers in New Zealand wheat and barley fields, here ordered by observations in wheat crops. The herbicides that were not ranked in the top ten for each crop respectively were included here for both of the crops for completeness.

Cases of resistance by species and risk scoring

The documented cases of herbicide-resistance for medium and high-risk species and all herbicide mode-of-action combinations are shown in Table 3 (a total of 46 medium and high-risk species). High-risk species were the eight grasses *Avena fatua, Avena sterilis, Digitaria sanguinalis, Echinochloa crus-galli, Lolium multiflorum, Lolium perenne, Phalaris minor, Poa annua* and eight broadleaf weeds *Amaranthus powellii, Chenopodium album, Erigeron bonariensis, Erigeron sumatrensis, Raphanus raphanistrum, Senecio vulgaris, Solanum nigrum, Stellaria media*. Considering our list of weed species known in wheat and barley in New Zealand, we can see that the number of weed species with herbicide resistance documented worldwide varies across HRAC herbicide groups B (34 species), C1 (21), G (16), O (13) and A (12) (Table 3). *Poa annua*, *Echinochloa crus-galli*, *Lolium* spp., *Erigeron sumatrensis*, *Raphanus raphanistrum* and *Avena fatua* have twenty or more recorded cases that occur in five or more unique mode-of-action groups each.

Table 3. Herbicide resistance risk scores and cases for herbicide mode-of-action groups and species. For weeds of wheat and barley in New Zealand we document the number of worldwide cases of herbicide resistance within different HRAC [26] groups. Data are sourced from the International Survey of Herbicide-Resistant Weeds [4] and concerns unique modes-of-action within reported cases of herbicide resistance. Species-risk and herbicide-risk are either low (1), medium (2), or high (3). Total cases and species per herbicide mode-of-action across worldwide cases [4] are presented at the bottom of the table. Symbols designate species with herbicide resistance cases detected in New Zealand wheat and barley *, or in other crops † [11,28]. Table footnotes report low-risk weeds.

**Species**	**Species Risk**	**A**	**B**	**C1**	**C2**	**C3**	**D**	**E**	**F1**	**F2**	**F3**	**F4**	**G**	**H**	**L**	**K3**	**K1**	**N**	**O**	**Z**	**Groups**	**Total Unique Cases**
**Herbicide Risk**		3	3	3	2	1	2	1	1	1	1	1	3	1	1	1	1	1	2	1		
Amaranthus powelii	3	0	8	8	0	0	0	0	0	0	0	0	0	0	0	0	0	0	0	0	2	16
*Avena fatua**	3	38	19	0	0	0	0	1	0	0	0	0	0	0	0	1	1	9	0	8	7	77
Avena sterilis	3	15	9	0	0	0	0	0	0	0	0	0	2	0	0	0	0	0	0	2	4	28
Brassica rapa	2	0	1	1	0	0	0	0	0	0	0	0	2	0	0	0	0	0	1	0	4	5
Bromus catharticus	2	0	0	0	0	0	0	0	0	0	0	0	1	0	0	0	0	0	0	0	1	1
Bromus diandrus	2	1	1	0	0	0	0	0	0	0	0	0	1	0	0	0	0	0	0	0	3	3
Bromus secalinus	2	0	2	0	0	0	0	0	0	0	0	0	0	0	0	0	0	0	0	0	1	2
Bromus sterilis	2	1	2	0	0	0	0	0	0	0	0	0	0	0	0	0	0	0	0	0	2	3
Capsella bursa-pastoris	2	0	6	2	0	0	0	0	0	0	0	0	0	0	0	0	0	0	0	0	2	8
Carduus nutans†	2	0	0	0	0	0	0	0	0	0	0	0	0	0	0	0	0	0	1	0	1	1
Chenopodium album†	3	0	7	41	0	0	0	0	0	0	0	0	0	0	0	0	0	0	1	0	3	49
Cirsium arvense	2	0	0	0	0	0	0	0	0	0	0	0	0	0	0	0	0	0	2	0	1	2
Critesion murinum	2	3	1	0	0	0	4	0	0	0	0	0	1	0	0	0	0	0	0	0	4	9

Convolvulus arvensis	2	0	0	0	0	0	1	0	0	0	0	0	0	0	0	0	0	0	0	0	1	1
Digitaria sanguinalis	3	7	3	3	0	0	0	0	0	0	0	0	0	0	0	0	0	0	0	0	3	13
Echinochloa crus-galli	3	14	16	6	11	0	0	0	0	0	0	1	1	0	2	3	0	2	7	0	10	63
Erigeron bonariensis	3	0	1	2	0	0	6	0	0	0	0	0	13	0	0	0	0	0	0	0	4	22
Erigeron sumatrensis	3	0	7	1	0	0	9	0	0	0	0	0	10	0	0	0	0	0	1	0	5	28
Fallopia convolvulus	2	0	2	2	0	0	0	0	0	0	0	0	0	0	0	0	0	0	0	0	2	4
Fumaria densiflora	2	0	0	0	0	0	0	0	0	0	0	0	1	0	0	0	1	0	0	0	2	2
Galium aparine	2	0	4	0	0	0	0	0	0	0	0	0	0	0	0	0	0	0	3	0	2	7
Lactuca serriola	2	0	5	0	0	0	0	0	0	0	0	0	1	0	0	0	0	0	1	0	3	7
Lamium amplexicaule	2	0	1	0	0	0	0	0	0	0	0	0	0	0	0	0	0	0	0	0	1	1
*Lolium multiflorum**	3	55	37	0	0	0	2	0	0	0	1	0	27	3	0	5	0	0	0	0	7	130
*Lolium perenne**	3	4	5	0	0	0	0	0	0	0	1	0	4	1	0	0	0	0	0	0	5	15
Persicaria maculosa†	2	0	1	4	0	0	0	0	0	0	0	0	0	0	0	0	0	0	0	0	2	5
Phalaris minor	3	9	4	0	0	0	0	0	0	0	0	0	0	0	0	0	0	0	0	0	2	13
Phalaris paradoxa	2	7	1	1	0	0	0	0	0	0	0	0	0	0	0	0	0	0	0	0	3	9
Plantago lanceolata	2	0	0	0	0	0	0	0	0	0	0	0	1	0	0	0	0	0	1	0	2	2
Poa annua†	3	3	10	19	0	0	2	0	0	0	1	0	7	0	0	0	7	1	4	0	9	54
Polygonum aviculare	2	0	0	1	0	0	0	0	0	0	1	0	0	0	0	0	0	0	0	0	2	2
Raphanus raphanistrum	3	0	9	1	0	0	0	0	3	0	0	0	1	0	0	0	0	0	6	0	5	20
Rumex acetosella	2	0	0	1	0	0	0	0	0	0	0	0	0	0	0	0	0	0	0	0	1	1

Rumex obtusifolius	2	0	1	0	0	0	0	0	0	0	0	0	0	0	0	0	0	0	0	0	1	1
Senecio vulgaris	3	0	2	13	0	1	0	0	0	0	0	0	0	0	0	0	0	0	0	0	3	16
Silene gallica	2	0	1	0	0	0	0	0	0	0	0	0	0	0	0	0	0	0	0	0	1	1
Solanum americanum†	2	0	0	0	0	0	2	0	0	0	0	0	0	0	0	0	0	0	0	0	1	2
Solanum nigrum†	3	0	0	11	0	0	3	0	0	0	0	0	0	0	0	0	0	0	0	0	2	14
Sonchus asper	2	0	5	1	0	0	0	0	0	0	0	0	0	0	0	0	0	0	0	0	2	6
Sonchus oleraceus	2	0	3	0	0	0	0	0	0	0	0	0	1	0	0	0	0	0	2	0	3	6
Spergula arvensis	2	0	1	0	0	0	0	0	0	0	0	0	0	0	0	0	0	0	0	0	1	1
Stellaria media†	3	0	20	1	0	0	0	0	0	0	0	0	0	0	0	0	0	0	2	0	3	23
Tripleurospermum inodorum	2	0	7	0	0	0	0	0	0	0	0	0	0	0	0	0	0	0	0	0	1	7
Urtica urens	2	0	0	1	0	0	0	0	0	0	0	0	0	0	0	0	0	0	0	0	1	1
Vicia sativa	2	0	1	0	0	0	0	0	0	0	0	0	0	0	0	0	0	0	0	0	1	1
Vulpia bromoides	2	0	0	2	0	0	1	0	0	0	0	0	0	0	0	0	0	0	0	0	2	3
**Total cases count**		157	203	122	11	1	30	1	3	0	4	1	74	4	2	9	9	12	32	10		683
**Total species count**		12	34	21	1	1	9	1	1	0	4	1	16	2	1	3	3	3	13	2		127

[a]We report ~~the~~ low risk weeds: *Achillea millefolium, Agrostis capillaris, Amaranthus deflexus, Amsinckia calycina, Aphanes arvensis, Arrhenatherum elatius, Avena barbata, Barbarea intermedia, Brassica napus, Bromus hordeaceus, Calandrinia compressa, Calandrinia menziesii, Cardamine flexuosa, Cerastium glomeratum, Chenopodiastrum murale, Cirsium vulgare, Crepis capillaris, Crepis setosa, Dactylis glomerata, Elytrigia repens, Erodium cicutarium, Erodium moschatum, Festuca rubra, Fumaria bastardii, Fumaria muralis, Fumaria officinalis, Gamochaeta coarctata, Gamochaeta purpurea, Geranium molle, Leontodon saxatilis, Lepidium didymum, Lotus pedunculatus, Lysimachia arvensis, Malva neglecta, Malva parviflora, Matricaria discoidea, Oxalis debilis, Oxalis latifolia, Phalaris aquatica, Phalaris canariensis, Ranunculus repens, Sherardia arvensis, Silene vulgaris, Sisymbrium officinale, Solanum sarrachoides, Stachys arvensis, Taraxacum officinale, Trifolium pratense, Trifolium repens, Veronica arvensis, Veronica persica, Vicia hirsuta, Vicia lathyroides, Viola arvensis, Vulpia myuros*

The cumulative risk scores shown in Fig [illegible] gives a higher score to weeds with resistance to multiple modes-of-action. Because of this species with high overall risk scores may have relatively few cases of resistance detected overall but their score is inflated by cases of resistance to multiple HRAC modes-of-action. For example *Lolium perenne* (15 cases in 5 modes-of-action) has a slightly lower risk score overall compared to its congener *Lolium multiflorum* (130 cases in 7 modes-of-action). *Chenopodium album* is an example of a relatively low scoring weed that has had 49 cases of resistance documented but within only HRAC modes-of-action (Fig 2, Table 3). Risks can be skewed toward certain herbicide modes-of-action, or species. Weeds with the ten highest cumulative scores are *Echinochloa crus-galli*, *Poa annua*, *Lolium multiflorum*, *Erigeron sumatrensis*, *Raphanus raphanistrum*, *Lolium perenne*, *Erigeron bonariensis*, *Avena fatua*, *Avena sterilis*, *Digitaria sanguinalis*. This shows that 7 out of 10 of the high risk species are grass-weeds. Grass-weeds are over-represented in global cases of resistance.

Fig 2. Number of cases of herbicide resistance (globally) by weed species and cumulative herbicide resistance risk scores. The Sum of Risk (cumulative risk) scores on the x-axis is the sum of the herbicide-risk × species-risk scores (from Table 3) for each HRAC herbicide mode-of-action group that had documented cases of resistance somewhere in the world. We also show the number of resistance cases documented in the world. The green dashed line shows the 10 cases needed for a species to be designated high risk (score 3). We distinguished the proportion of the risk and resistance cases that matched with the high-use HRAC mode-of-action groups (A, B, C1, F1, G, K3 & O) used by wheat and barley farmers in New Zealand (light-green for risk, and grey for cases).

Discussion

We present evidence about the propensity of individual weed species to develop resistance in a curated list (Table S1) of weed species known to occur in New Zealand wheat and barley fields [22]. The ten highest cumulative risk scores, in order *Echinochloa crus-galli, Poa annua, Lolium multiflorum, Erigeron sumatrensis, Raphanus raphanistrum, Lolium perenne, Erigeron bonariensis, Avena fatua, Avena sterilis and Digitaria sanguinalis*. We expect to see novel instances of resistance in common weeds with moderate-to-high risk scores (in order): *Erigeron* spp., *Raphanus raphanistrum, Chenopodium album, Senecio vulgaris, Phalaris* spp., *Bromus diandrus, Sonchus oleraceus, Solanum nigrum* and *Persicaria maculosa*. Species to species differences in distribution, abundance and phenology (e.g. germination timing) will mean that our risk scores do not capture field level differences in selection pressure from farmer herbicide applications in wheat in barley. As such, emerging grass weeds *Echinochloa crus-galli and Digitaria sanguinalis* are identified as high-risk despite their currently limited distribution. By being aware of all the weed species that are high-risk we should improve detection of resistance cases in future.

A review of the New Zealand literature shows only two reports of resistant species in wheat and barley [7,11]A review of the New Zealand literature shows only two reports of resistant species in wheat and barley [7,11]. This contrasts with the high numbers of resistance cases seen worldwide for these crops (77 cases in wheat, and 30 in barley) [4]. One might expect cases to be reported quickly, given that selection for rare mutations that confer resistance is infrequent but instantaneous. But surviving individuals and progeny may take a few years to increase to detectable levels (in a field) under continuous selection pressure [30]. In New Zealand resistance was detected in 2014 for *Avena*

fatua to acetyl coenzyme-A carboxylase (group A, ACCase)[31], and in 2017 for ryegrasses (*Lolium perenne* and *L. multiflorum*) to ACCase and acetolactate synthase targeting herbicides (ALS herbicide, group B) [31,32]. A 1996 report of *Stellaria media* resistance to chlorsulfuron and tribenuron (group B) in an oat crop may indicate elevated risk given the shared agronomic practices between these cereal crops [33]. The resistant weeds previously detected in New Zealand cereals *Lolium* spp., and *Avena* spp. and *Stellaria media*, are therefore likely to continue to be observed. It seems likely that farmers are under-reporting resistance cases perhaps because alternative weed control measures can keep problems manageable. In the absence of a systematic approach, little is known about the spatial and temporal patterns of herbicide resistance development in New Zealand.

Estimating the overall prevalence of herbicide resistance in all the major farming sectors in New Zealand could cost $1-3 million NZD depending on sampling rates [11]. An obvious concern is that detection rates for herbicide-resistant weeds will necessarily underestimate the true rate, given that surveyors may miss individual weed species, resistant plants, or seeds during farm visits [11] or they could miss cases by screening for the wrong herbicides. Surveys have been initiated for the arable sector in New Zealand's Canterbury region where wheat and barley are important crops. They will sample about 20% of ca. 800 arable farms at an estimated cost of ca. $154,000. Given these high survey costs, it is important to take steps to improve detection rates, the high-risk species identified here should be targeted during surveys. Without this list we could be biased toward a smaller number of known problem weeds, such as *Lolium* spp. and *Avena fatua*.

The Moss protocol relies heavily on a herbicide-risk score (rank low, medium and high). We deviated from their approach slightly. This was necessary in part because the number of cases for different herbicide groups has increased since the Moss article was published. A case in point is our decision to include glyphosate as a high-risk herbicide. As recently as 2006 glyphosate was ranked as amongst the least likely to select for resistance [29], and is recognized as medium-risk by Moss. We ranked glyphosate (group G; Table 1) as high risk because worldwide the number of species showing resistance has increased to 47 cases, similar to group A (48 cases) which is universally regarded as high-risk. Group A herbicides were ranked as high-risk in the Moss protocol. Two cases of glyphosate resistance are known from New Zealand [7,11]. If we had used the 10% threshold groups, A and G would be medium risk and species scores would have changed, but the top five ranked species would have remained the same. We are aware that herbicide-risk is not just a function of the number associated cases of resistance. More complete risk assessments would ideally factor in the herbicide volumes used, years of product use, spatial extent and number of the applications, as well as the abundance of high-risk weeds in the areas treated.

Herbicide usage data in New Zealand have rarely been quantified, and only roughly via indirect sales data numbers and expert elicitation [34,35]. The ProductionWise® system is used to record the on-farm use of chemical inputs and other information. This system (and similar tools) are valuable for ensuring farmer compliance with record-keeping regulations, supporting farmer decision-making, and guiding herbicide resistance prevention efforts. It also serves to capture industry-wide behaviour regarding agrichemical use which is how we have used it in this case. Herbicide resistance risk is influenced by the other crops in the rotation, and temporal and spatial differences in weed composition. The relatively low rates of herbicide resistance detected in the New Zealand arable sector may be a consequence of mixed-crop rotation systems. It is not uncommon to include a 1-3

year pasture rotation, and a complex crop sequence, for example, winter wheat, spring-sown peas or linseed, winter wheat or barley, followed by grass, and oilseed rape and back to winter wheat [36]. Cases of herbicide resistance we observe now only partly reflect current herbicide use and may, in fact, implicate historic selection by herbicides that have fallen out of favour. We think advanced record-keeping tools like this and related decision support systems have real potential to improve outcomes for farmers and scientists.

Within New Zealand wheat and barley fields herbicide use and risk of resistance are greatest within HRAC groups (in order) O, B, G, A and C1. Surprisingly, there have been few cases of resistance to ALS-inhibitors (B) and synthetic auxins (O) documented to date given that they have been the most commonly applied herbicides for more than 20 years [37,38]. Field observations show that broadleaf weeds are rarer in the wheat fields prior to harvest compared to grass weeds, but survivors should be tested for herbicide resistance. Group B herbicides have been implicated in a large number of resistance cases in both broadleaf and grass weeds, including 34 species known to occur in wheat and barley in NZ (Table 3). Groups F1 and K3 have a relatively low risk of developing resistance based on historical occurrences even though they are highly used in New Zealand.

Examining our herbicide use information in the context of published herbicide evolution models can provide important insights. Models based on dryland wheat systems and the weed *Lolium rigidum* [39] showed that resistance rate evolution was not slowed by simple herbicide rotations (i.e. annual with few herbicides). Importantly the use of soil-applied herbicides, particularly trifluralin (group K1), full-rate mixes of herbicides, and complex 8-year long rotations were shown to delay resistance evolution by years, and in some scenarios by decades [39]. There is a chance that resistance cases in soil-applied pre-emergent herbicides are under-reported compared to post-emergent ones (they are harder to test). For now, resistance evolution in those herbicides appears to be slower. Trifluralin did not feature amongst the most common herbicides in the farmer herbicide use data we obtained for 2017 and 2018 (<0.5% of field applications); the only frequently used pre-emergent herbicides were flufenacet and terbuthylazine. Farmers should be informed about the high-risk species and herbicide combinations, so as to avoid high-risk behaviors, or at least keep an eye out for problems they will select for. Farmer decision support platforms and research and extension efforts in New Zealand should emphasize mechanical and cultural control measures, the use of soil-applied pre-emergent herbicides, full-strength label-rate herbicide mixtures, crop rotation to utilize herbicides otherwise unavailable and herbicide rotations of key active ingredients to achieve maximum control and to reduce the rate at which herbicide resistance evolves in weed populations.

Conclusions

A European protocol [20], designed primarily to assist in herbicide authorization procedures, was adapted to assess the risk of herbicide resistance evolving in 100 different weed species known to occur in New Zealand wheat and barley crops. More than half the weeds weeds (55) we assessed were low-risk, 29 were medium-risk and 16 high-risk. The 10 species posing the highest resistance risk were: *Echinochloa crus-galli, Poa annua, Lolium multiflorum, Erigeron sumatrensis, Raphanus raphanistrum, Lolium perenne, Erigeron bonariensis, Avena fatua, Avena sterilis* and *Digitaria sanguinalis*. To provide important context we also report on herbicide use patterns in New Zealand

wheat and barley fields. We are planning extensive surveys in New Zealand to detect new cases of herbicide resistance. The risk assessment outlined in this paper will enable us to prioritise those weeds identified as posing a high resistance risk and, consequently, make better use of available resources. The risk assessment procedure as described in this paper has the potential to be a useful tool for evaluating the risk of herbicide resistance in a wide range of different weed species in other countries too.

Acknowledgments

Ian Heap's International survey of herbicide resistant weeds was an important resource for this article. This work was supported by the Ministry of Business, Innovation and Employment [grant number C10X1806] to AgResearch Ltd.: "Improved weed control and vegetation management to minimize future herbicide resistance." Shona Lamoureaux, Phil Hulme, Trevor James, Hossein Ghanizadeh, Ian Heap and Graeme Bourdôt provided helpful comments on a draft.

References

1. Oerke E-C. Crop losses to pests. J Agric Sci. 2006;144: 31–43.
2. Stephenson GR. Pesticide Use and World Food Production: Risks and Benefits. Expert Committee on Weeds - Comité d'experts en malherbologie. Banff, Canada: Canadian Weed Science Society; 2000. pp. 9–15. doi:10.1021/bk-2003-0853.ch015
3. Thorne ME, Young FL, Pan WL, Alldredge JR. No-till spring cereal cropping systems reduce wind erosion susceptibility in the wheat/fallow region of the Pacific Northwest. J Soil Water Conserv. 2003; 9.
4. Heap I. International survey of herbicide resistant weeds - weedscience.org. 2020 [cited 25 Jan 2020]. Available: http://www.weedscience.org/
5. Baucom RS. The remarkable repeated evolution of herbicide resistance. Am J Bot. 2016;103: 181–183. doi:10.3732/ajb.1500510
6. Powles SB, Yu Q. Evolution in Action: Plants Resistant to Herbicides. Annu Rev Plant Biol. 2010;61: 317–347. doi:10.1146/annurev-arplant-042809-112119
7. Ghanizadeh H, Harrington KC. Herbicide resistant weeds in New Zealand: state of knowledge. N Z J Agric Res. 2019; 1–12. doi:10.1080/00288233.2019.1705863
8. Millner JP, Roskruge NR. The New Zealand arable industry. In: Dymond JR, editor. Ecosystem services in New Zealand – conditions and trends. Manaaki Whenua Press, Landcare Research; 2013. pp. 102–114.

9. Watson E. The highest wheat yield. In: Guinness World Records [Internet]. 17 Feb 2017 [cited 13 May 2020]. Available: https://www.guinnessworldrecords.com/world-records/highest-wheat-yield

10. Darling W. The highest barley yield. In: Guinness World Records [Internet]. 23 Jan 2015 [cited 13 May 2020]. Available: https://www.guinnessworldrecords.com/world-records/68919-highest-barley-yield

11. Buddenhagen CE, Gunnarsson M, Rolston P, Chynoweth RJ, Bourdot G, James TK. Costs and risks associated with surveying the extent of herbicide resistance in New Zealand. N Z J Agric Res. 2019;0: 1–19. doi:10.1080/00288233.2019.1636829

12. Broster JC, Koetz EA, Wu H. Herbicide resistance frequencies in ryegrass (*Lolium* spp.) and other grass species in Tasmania. Plant Prot Q. 2012;27: 36–42.

13. Broster JC, Koetz EA, Wu H. Herbicide resistance levels in annual ryegrass (*Lolium rigidum* Gaud.) in southern New South Wales. Plant Prot Q. 2011;26: 22–28.

14. Broster JC, Koetz EA, Wu H. A survey of southern New South Wales to determine the level of herbicide resistance in brome grass and barley grass populations. In: Zydenbos SM, editor. Seventeenth Australasian Weeds Conference. Christchurch, New Zealand; 2010. pp. 274–277.

15. Owen MJ, Goggin DE, Powles SB. Non-target-site-based resistance to ALS-inhibiting herbicides in six *Bromus rigidus* populations from Western Australian cropping fields. Pest Manag Sci. 2012;68: 1077–1082. doi:10.1002/ps.3270

16. Owen MJ, Martinez NJ, Powles SB. Herbicide resistance in *Bromus* and *Hordeum* spp. in the Western Australian grain belt. Crop Pasture Sci. 2015;66: 466. doi:10.1071/CP14293

17. Owen MJ, Martinez NJ, Powles SB. Multiple herbicide-resistant wild radish (*Raphanus raphanistrum*) populations dominate Western Australian cropping fields. Crop Pasture Sci. 2015;66: 1079. doi:10.1071/CP15063

18. Menchari Y, Camilleri C, Michel S, Brunel D, Dessaint F, Le Corre V, et al. Weed response to herbicides: regional-scale distribution of herbicide resistance alleles in the grass weed *Alopecurus myosuroides*. New Phytol. 2006;171: 861–874. doi:10.1111/j.1469-8137.2006.01788.x

19. Beckie HJ, Thomas AG, Stevenson FC. Survey of herbicide-resistant wild oat (*Avena fatua*) in two townships in Saskatchewan. Can J Plant Sci. 2002;82: 463–471. doi:10.4141/P01-067

20. Moss S, Ulber L, den Hoed I. A herbicide resistance risk matrix. Crop Prot. 2019;115: 13–19. doi:10.1016/j.cropro.2018.09.005

21. PP 1/213 (4) Resistance risk analysis. EPPO Bull. 2015;45: 371–387. doi:10.1111/epp.12246

22. Bourdôt GW, Hurrell GA, Saville DJ. Weed flora of cereal crops in Canterbury, New Zealand. N Z J Crop Hortic Sci. 1998;26: 233–247. doi:10.1080/01140671.1998.9514059

23. Rolston MP, Archie WJ, Reddy K, Dastgheib F. Grass weed control and herbicide tolerance in cereals. N Z Plant Prot. 2003;56: 220–226. doi:10.30843/nzpp.2003.56.6095

24. Kon KF, Follas GB, James DE. Seed dormancy and germination phenology of grass weeds and implications for their control in cereals. N Z Plant Prot. 2007;60: 174–182. doi:10.30843/nzpp.2007.60.4597

25. Breitwieser I, Brownsey PJ, Heenan PB, Nelson WA, Wilton AD. Flora of New Zealand Online. 2010 [cited 15 Feb 2020]. Available: http://www.nzflora.info/

26. HRAC. Global Classification Lookup. In: Herbicide Resistance Action Committee [Internet]. 2020 [cited 20 Jan 2020]. Available: https://hracglobal.com/tools/classification-lookup

27. ProductionWise – ProductionWise by Grain Growers. 2020 [cited 25 Feb 2020]. Available: https://productionwise.co.nz/

28. R Core Team. R: A Language and Environment for Statistical Computing. Vienna, Austria: R Foundation for Statistical Computing; 2020. Available: https://www.R-project.org/

29. Wickham H. ggplot2: Elegant Graphics for Data Analysis. Springer-Verlag New York; 2016. Available: http://ggplot2.org

30. Jasieniuk M, Brûlé-Babel AL, Morrison IN. The Evolution and Genetics of Herbicide Resistance in Weeds. Weed Sci. 1996;44: 176–193. doi:10.1017/S0043174500093747

31. Harrington KC, Chynoweth RJ. Have wild oats in Canterbury become resistant to herbicides. N Z Plant Prot. 2014;67: 331–331. doi:10.30843/nzpp.2014.67.5778

32. Gunnarsson M, James TK, Chynoweth RJ, Rolston MP. An evaluation of the resistance of annual and perennial ryegrass to herbicides. N Z Plant Prot. 2017;70: 165–170.

33. Bourdôt GW. The status of herbicide resistance in New Zealand weeds. Pestic Resist Prev Manag N Z Plant Prot Soc Rotorua N Z. 1996; 81–89.

34. NIWA Library catalogue › Details for: Patterns of pesticide use in New Zealand. Part 2, South Island 1986-1989 /. [cited 7 Jan 2020]. Available: https://library.niwa.co.nz/cgi-bin/koha/opac-detail.pl?resultid=2&biblionumber=272431&query_desc=

35. New Zealand, ACVM Group. ACVM registration information requirements for provisional registrations. Wellington, N.Z.: Ministry of Agriculture and Forestry; 1999.

36. Hampton JG, Rolston MP, Pyke NB, Green W, Lincoln A. Ensuring the long term viability of the New Zealand seed industry. Agron N Z. 2012; 12.

37. Holland PT, Rahman A. Review of trends in agricultural pesticide use in New Zealand. Ministry of Agriculture and Forestry Wellington,, New Zealand; 1999.

38. Manktelow D, Stevens P, Walker J, Gurnsey S, Park N, Zabkiewicz J, et al. Trends in pesticide use in New Zealand: 2004. Rep Minist Environ Proj No SMF4193. 2005.

39. Busi R, Powles SB, Beckie HJ, Renton M. Rotations and mixtures of soil- applied herbicides delay resistance. Pest Manag Sci. 2019; ps.5534. doi:10.1002/ps.5534

Table S1. The full list of weed species considered in our risk assessment for herbicide resistance in New Zealand Wheat and Barley crops. The list is derived from Bourdôt et al. [22], with additions from literature [23,24], expert knowledge and field observations made in January (late summer) of 2019 and 2020. Common name (in New Zealand) and family name are indicated. Nomenclature follows the New Zealand Flora [25].

Figure 1

Click here to access/download;Figure;Fig1.tif

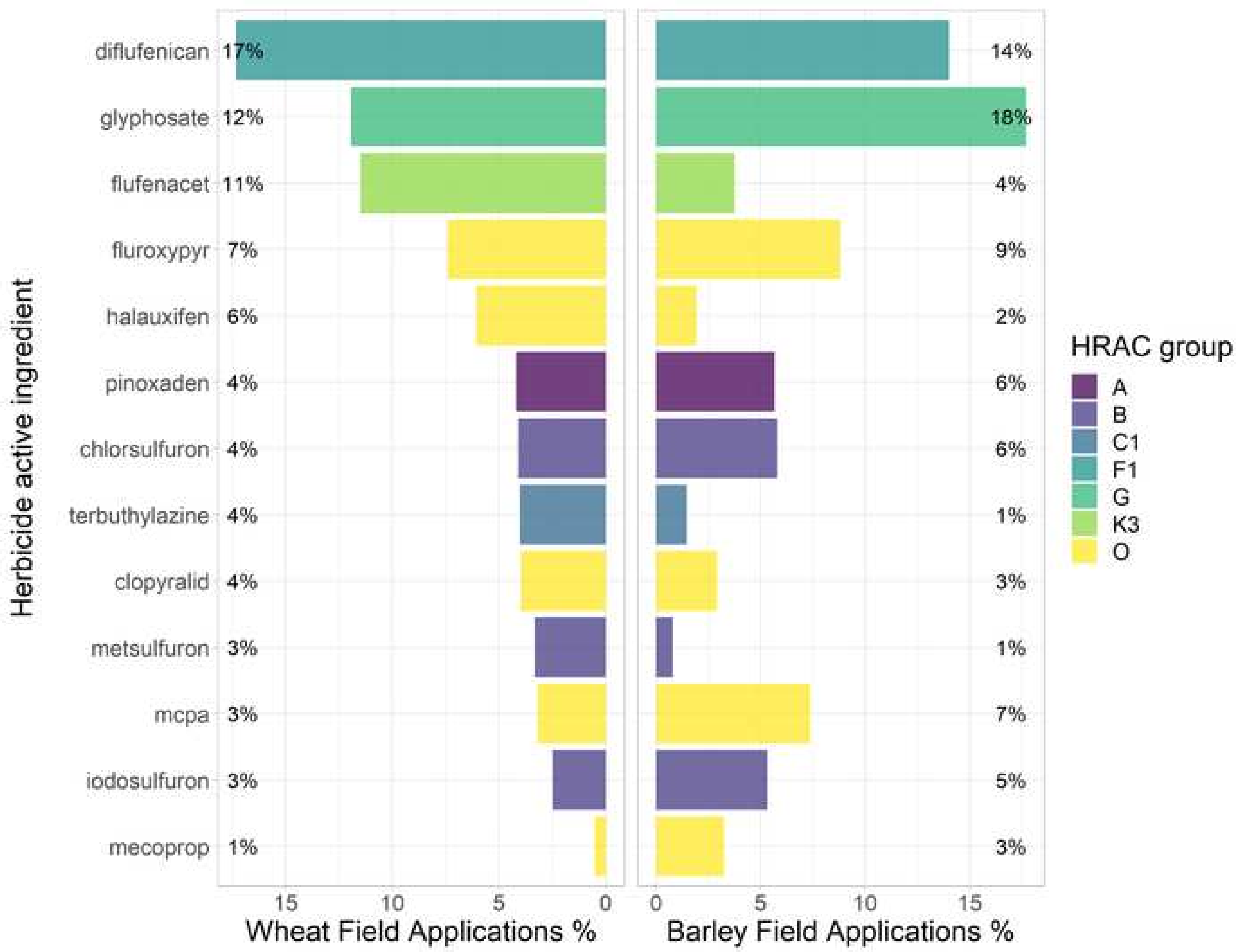

Figure 2

Click here to access/download;Figure;Fig2.tif

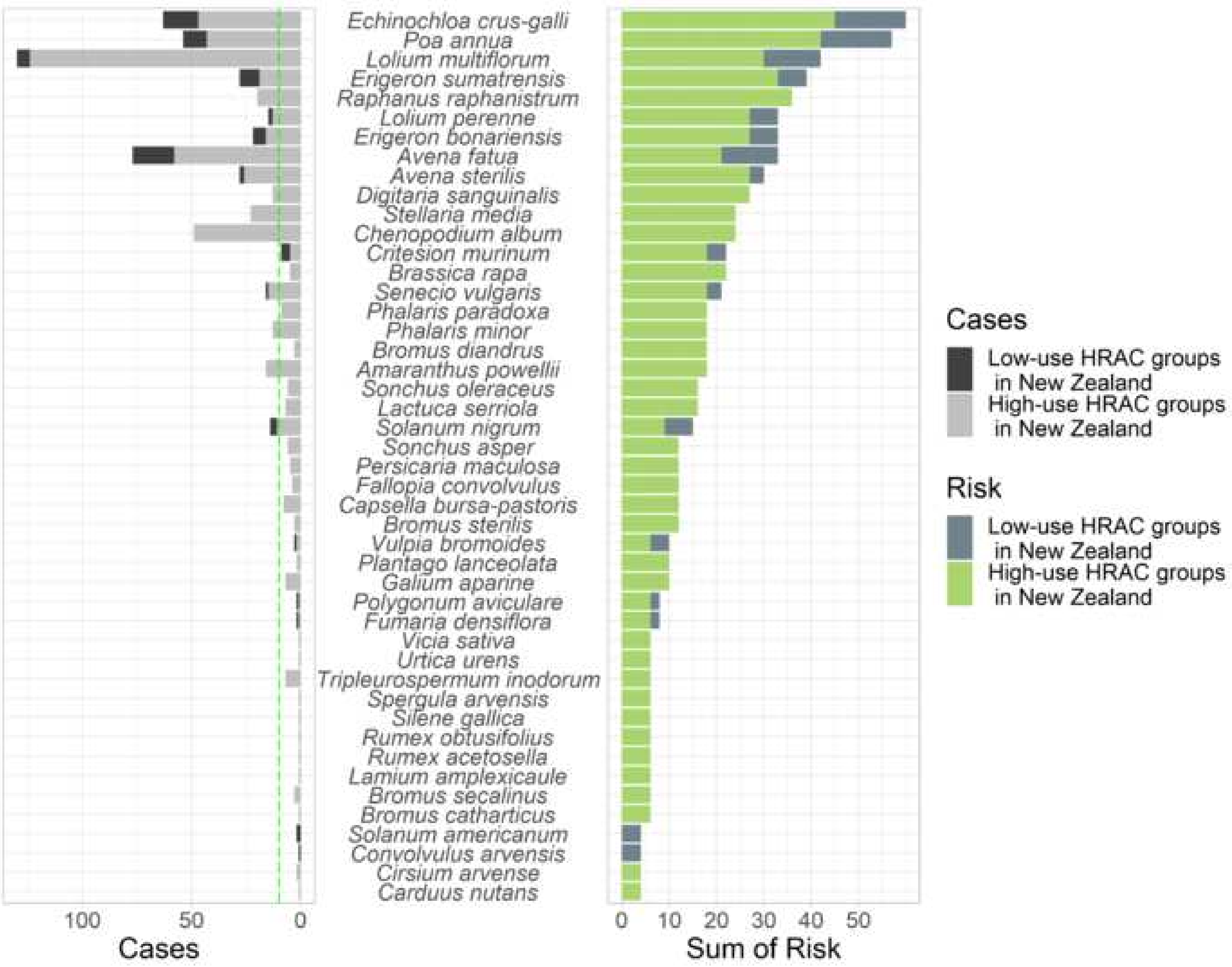

Click here to access/download
Supporting Information
S1_File.pdf

A herbicide resistance risk assessment for weeds in wheat and barley crops in New Zealand

Zachary Ngow[1*¶], Richard J. Chynoweth[2&], Matilda Gunnarsson[2&], Phil Rolston[2&], Christopher E. Buddenhagen[1*¶]

[1] AgResearch Ltd., Hamilton, New Zealand

[2] Foundation for Arable Research, Christchurch, New Zealand

* Corresponding Authors: E-mail: zacharyngow@gmail.com, E-mail: chris.buddenhagen@agresearch.co.nz

[¶] These authors contributed equally to this work.

[&] These authors also contributed equally to this work.

Abstract

We estimated the risk of selecting for herbicide resistance in 101~~0~~ weed species known to occur in wheat and barley crops on farms in New Zealand. A protocol was used that accounts for both the risk that different herbicides will select for resistance and each weed's propensity to develop herbicide resistance based on the number of cases worldwide. ~~We estimated the risk of selecting for herbicide resistance in 100 weed species known to occur in New Zealand wheat and barley farms using a protocol that accounts for the risk that different herbicides will select for resistance, and each weed's propensity to develop herbicide resistance based on the cases worldwide.~~ To provide context we documented current herbicide use patterns. Most weeds (55) were low-risk, 30~~29~~ were medium-risk and 16 high-risk. The top ten scored weeds were *Echinochloa crus-galli*, *Poa annua*, *Lolium multiflorum*, *Erigeron sumatrensis*, *Raphanus raphanistrum*, *Lolium perenne*, *Erigeron bonariensis*, *Avena fatua*, *Avena sterilis* and *Digitaria sanguinalis*. Seven out of ten high-risk weeds were grasses. The most used herbicides were synthetic auxins, an enolpyruvylshikimate-phosphate synthase inhibitor, acetolactate synthase (ALS) inhibitors, carotenoid biosynthesis inhibitors, and long-chain fatty acid inhibitors. ALS-inhibitors ~~contributed to~~were assessed as posing the greatest ~~the~~ risk for more species than other mode~~s~~-of-action. Despite pre-emergence herbicides being known to delay resistance, New Zealand farmers only applied flufenacet and terbuthlazine with high frequency.~~Pre-emergent herbicides are known to delay resistance, flufenacet and terbuthylazine were the only active ingredients in this class favoured by New Zealand farmers.~~ Based on our analysis, surveys for herbicide-~~-~~resistant species should focus on the high-risk species we identified. Farmer extension efforts in New Zealand should address resistance evolution in cropping weeds.

Introduction

A worldwide analysis suggests that weeds have the highest potential to cause yield losses in major crops including wheat and barley where they account for ~~average~~ potential losses of 23% [1,2]. In response to this threat to production, farmers have come to rely on a mix of cultural and chemical control methods. Herbicidal weed control is favoured as it is cost-effective providing a three or four-fold economic return [2] and is a key element in the implementation of conservation tillage systems (including direct drilling) which reduce soil erosion [3]. However, intensive herbicide use is known to select for resistant individuals in weed populations, eroding herbicide effectiveness as these weeds escape control [4–6]. Globally farmers are addressing similar suites of weed species in any given crop, and some appear to have a greater propensity to develop resistance, with repeated convergent evolution of resistance being documented in the International Survey of Herbicide Resistant Weeds [4] and the scientific literature generally [5,6]. Over the last 40 years a total of ~~25 cases of~~14 species of herbicide resistant weeds have been documented in New Zealand, with 12 ~~of~~ le of resistance documented ~~which were~~ in arable crops including maize, peas, oats, wheat, and barley [7]. In any given year >50% of New Zealand arable production areas are under wheat (~45000 ha) and barley (~55000 ha) rotations [8]. Production levels are high, with farmers in New Zealand obtaining world record wheat and barley yields in ~~. Yields for wheat regularly pass 10 tonnes per hectare in New Zealand, and in 2016 an Ashburton farmer achieved the world record for wheat~~ 2017 and 2015, respectively ~~yield with 16.79 tonnes per hectare~~ [9,10]~~[9]~~.~~[10]~~.

An examination of weed science publications shows that our knowledge about herbicide resistance cases has developed mostly via unsystematic detection globally. It reflects the varying effort, scientific input, and methods of detection; definitely this is the case in New Zealand [7,11]. This makes sense since farmers and herbicide companies do not necessarily report resistance cases to scientists, and even if they do, not all cases are likely to get published. What's more, ad-hoc reporting may reflect strong biases towards a small number of the most problematic weeds. With some notable exceptions, particularly in cropping systems in Australia [12–17]~~[11–16]~~~~[9–14]~~, systematic surveys to detect herbicide resistance cases are rare, and often focus on one or two problematic species in a given crop, for example, *Alopecurus myosuroides* Huds. in French wheat fields [18]~~[17]~~~~[15]~~ or *Avena fatua* L. in two Canadian townships [19]~~[18]~~~~[16]~~. Systematic surveys may be rare because of the cost, a lack of specific pathways for reporting [11]~~[10]~~~~[8]~~, and industry perceptions about the importance of resistance. Surveyors should ideally be open to the discovery of completely novel cases while also being aware of those that are most at risk of developing resistance.

Here we adapt a recently published risk assessment protocol [20]~~[19]~~~~[17]~~ to identify those weeds most at risk of developing resistance in New Zealand, given their occurrence in wheat and barley fields, and their prior record of resistance in wheat and barley fields elsewhere in the world. This Moss et al. protocol [20]~~[19]~~~~[17]~~ set out to assess resistance risk as part of a pesticide authorization process in Europe, based on a European Plant Protection Organization (EPPO) protocol, originally developed in 1999 [21]~~[20]~~~~[18]~~. They present a quantitative risk matrix using both herbicide-risk (some herbi-cides pose a higher risk than others) and species-risk (some weed species are more resistance-prone than others), with an optional score modifier designed to account for agronomic management practices that may reduce the risk.~~They present a quantitative risk matrix using ranked herbicide-risk and species-risk with an optional score modifier designed to account for agronomic management practices that may reduce the risk.~~ We took advantage of a unique data set about herbicide use in wheat and barley fields in New Zealand to place our risk assessment into context, and construct a framework for herbicide resistance surveys and extension efforts in the New Zealand cropping industry. This risk assessment is on an industry-wide scale informed by anonymized herbicide application data from wheat and barley fields. ~~Field and farm scale risk is not assessed~~Risks were not assessed at the scale of individual farms and fields, this requires detailed information about herbicide timing, mixtures and rotations, and their interactions with weed biology, crop rotations and other cultural practices. All the high-risk weeds identified here should be targeted in surveys designed to detect herbicide-resistant weeds.

Materials and Methods

Weed list

We generated a list of potential target weeds from wheat and barley crops in New Zealand. This was primarily sourced from the Bourdôt et al. 1998 weed survey in New Zealand (Canterbury region) wheat and barley fields [22]~~[21]~~~~[19]~~. The Canterbury and northern Otago regions contain more than 75% of all the wheat and barley grown in New Zealand [8]~~[20]~~. We expanded the weed species list to include species known to occur in wheat and barley fields in the wider New Zealand context.

Grasses and some broadleaf genera were not identified to species by Bourdôt et al. [22][21][19], hence we took steps to address this gap and other omissions by using other literature [23,24][22,23][21,22], expert knowledge and field observations made in January (late summer) of 2019 and 2020. Species nomenclature follows the New Zealand Flora and taxonomic authorities are listed in Table Table S12. [25][24][23]. Subspecies were not distinguished, and taxa were considered by us only at the species level (e.g. *Avena sterilis* subsp. *ludoviciana* (Durieu) M.Gillet & Magne was treated as *Avena sterilis* L.*Arrhenatherum elatius* ssp. *bulbosum* (Willd.) Schübl. & G. Martens was considered as *Arrhenatherum elatius* (L.) P. Beauv. ex J. Presl & C. Presl).

Ranking herbicide groups by resistance cases

We ranked Herbicide Resistance Action Committee (HRAC) legacy herbicide mode of action (MoA) groups legacy herbicide groups by the number of resistance cases documented by the International Survey of Herbicide Resistant Weeds [4] to obtain an estimate of what Moss et al. called the "inherent risk" of the herbicide [20][19][17]. The Moss et al. protocol [20][19][17] (henceforth the "Moss protocol") considers the inherent risk to relate to the total number of cases of resistance reported in the International Survey of Herbicide Resistant Weeds [4] for each legacy HRAC group MoA [26][25][24], with risk scores of 1, 2 or 3 given for low, medium or high risk respectivelywith risk scores of 1, 2 or 3 given for low, medium or high respectively. We set the threshold for high-risk at >9% of recorded cases, which captures the original high-risk categories identified in the Moss protocol, but now places group G (glyphosate) in the high category. Moss et al. used the 10% threshold for high-risk herbicides. With group A having 48 cases and group G herbicides having 47 cases we chose to place the two groups in the same risk category, with a difference in the numbers of cases of just worldwide we believe they are indistinguishable from the data. The alternative is to use the same threshold as in the Moss protocol, but this would result in group A and G being medium risk, which fails to capture the high-risk status of group A herbicides. There are different ways we can reasonably set risk thresholds, which are discussed later. Remaining ranking thresholds were not changed: medium risk 5-9%, low risk 1-5% and very low risk <1%. Low-risk and very low risk are both scored with a oneas '1'.

Herbicide use

The most current recent (2017-2018) data on herbicide usage trends in New Zealand were sourced from the ProductionWise® [27][26][25] platform and aggregated by active ingredient. This consisted of data entered by farmers about herbicide use in 5026 barley and 7647 wheat fields. Approximately 900 arable farmers have registered to use the platform, but anonymization was complete, with no unique identifiers for farms or fields. Farmers recorded every spray event (by herbicide product) in their fields. For example, an individual active ingredient used three times in a field is recorded three times. Products with multiple active ingredients were recorded as independent applications. Counts of herbicide use in fields were summarized by active ingredient and legacy HRAC [26][25][24] herbicide mode-of-action, from product label information. Relative rates of use by mode-of-action were quantified and characterized as very high (>20% of all application instances), high (>10%), moderate (>1%), low (~1%), extremely low (<1%) and nonexistent (0%). The most

used herbicides for each crop were characterized by weighting active ingredient amounts and the number of fields they were applied to.

Cases of resistance by taxon and risk scoring

We assume that the best way to predict resistance in a weed taxon species to any given herbicide (by HRAC group) is proportional to the number of documented cases of herbicide resistance in the same taxon, given the use of the same herbicide type in New Zealand. To calculate Moss's "inherent" species-risk scores we used the global number of resistance cases from the International Survey of Herbicide Resistant Weeds [4]. Cases are defined by the International Survey of Herbicide Resistant Weeds as unique combinations of weed species and HRAC herbicide mode-of-action (species x site of action). –To obtain the 'high', 'medium' and 'low' risk scores as used in the —Moss protocolTo obtain the high, medium and low scores like those in the Moss protocol [20][19][17], we designated ≥10 cases as high risk (score=3), < 10 as moderate (score=2) and no cases recorded as low risk (score=1). We assessed overall species-risk as the sum of the herbicide-risk multiplied by the "inherent" species-risk We assessed overall species-risk as the sum of the herbicide-risk multiplied by the "inherent" species-risk [20][19][17] combined for all relevant HRAC MoA herbicide groups, but onlyonce for each HRAC herbicide group, but only where species had cases of resistance documented somewhere in the world (Table S1Table 1). We include all cases of resistance for each weed species, rather than restricting our focus to cases from wheat and barley, because we are interested in a species propensity to develop resistance to a herbicide group. For example, the high-risk species *Chenopodium album* L. has more than 10 documented cases of resistance giving it a species score of 3. Then we consider the herbicide-risk scores for those herbicides where *Chenopodium album* has evolved resistance somewhere in the world. There were cases in two high-risk herbicide groups B and C1 (each with a herbicide score of 3), and one medium-risk group O (herbicide score of 2). The species and herbicide scores are multiplied and summed (3×3) + (3×3) + (3×2) = 24. We distinguished cases that were in herbicide groups highly-used (or not) by wheat and barley farmers in New Zealand.

Table 1. Herbicide resistance risk scores and cases for herbicide mode-of-action groups and species. For weeds of wheat and barley in New Zealand we document the number of worldwide cases of herbicide resistance within different HRAC [26] groups. Data are sourced from the International Survey of Herbicide-Resistant Weeds [4] and concerns unique modes-of-action within reported cases of herbicide resistance. Species-risk and herbicide-risk are either low (1), medium (2), or high (3). Total cases and taxa per herbicide mode-of-action across worldwide cases [4] are presented at the bottom of the table. Table footnotes report low-risk weeds.a

The summed (cumulative) scoring method described above is not used in the European Moss protocol because its purpose was to regulate herbicide product use [20][19][17]. In contrast, we wanted to determine the risk that different herbicides will select for resistance in weed species known to occur in New Zealand's wheat and barley fields. Ultimately we hope to inform sector stakeholders about risk, and to improve herbicide resistance detection. We adapted their protocol to score species-risk in an industry-wide assessment. Unlike Moss et al. [20][19][17] we used an explicit threshold (though arbitrary) to to determine species-risk scores based on the number of cases of resistance worldwide, but we think this set species-risk scores based on the number of cases (see methods) of

~~resistance but we think this~~ approach produces credible risk estimates in the light of current knowledge. We examined 101~~0~~ species and ended up with 46~~5~~ ‘high’ and ‘medium’ resistance risk species, many more than Moss et al ~~45 high and medium risk species, much more than Moss et al.~~ (they scored an example list of only 13 high and medium risk taxa). The Moss protocol also used score modifiers that take into account resistance management practices including the use of non-chemical control measures~~The Moss protocol also used score modifiers that take into account resistance management practices~~. We did not use the score modifiers since these vary from field to field and our objective was slightly different. We acknowledge that actual risk of resistance development is determined mostly by the frequency and type of herbicide applied (selection pressure) interacting with characteristics of weed biology, distribution and abundance ~~We did not use the score modifiers since these vary from field to field. We acknowledge that actual risk of resistance development is determined mostly by the frequency and type of herbicide applied (selection pressure) interacting with features of weed biology, distribution and abundance~~ [5]. All graphs were created in R using the ggplot2 package [28,29].

Results

Weed list

Our weed list contained 69 species from the original Bourdôt et al. article [22]~~[21][19]~~. An additional 32~~1~~ species were added ~~Thirty-one more species were added~~ based on field observation, expert opinion and relevant literature [23,24]~~[22,23][21,22]~~, resulting in a total of 101~~0~~ weed species for consideration~~leading to a total 100 weeds considered~~. *Digitaria sanguinalis*, *Echinochloa crus-galli*, *Erigeron* spp. and *Raphanus raphanistrum* are notable emerging weeds, so they were included. Some taxa noted by Bourdôt were resolved to species, such as *Trifolium* spp., which became *Trifolium repens* and *Trifolium pratense*. ~~Sixteen high-risk and 29 moderate-risk species are shown in Table S1Table 1, and the other low-risk species are discussed below.~~ A full list of weeds considered for New Zealand wheat and barley crops is displayed in ~~Table S2~~Table S1.

Ranking herbicide groups by resistance cases

Herbicide mode-of-action risk rankings are displayed (~~Table 1~~Table 1~~2~~), arranged from high risk to very low risk. This resembles the table of Moss et al., [20] with a notable exception in that group G is raised to high risk. We designated HRAC groups B, C1, A, G as high-risk (>9% of recorded cases), O, D, C2 as moderate (5-9%), E, K1, K3, N F, Z as low (1-5%) and C3, F1, H, L as very low risk (<1%).

~~Table 1~~Table 1~~2~~. Herbicide mode-of-action groups ranked by resistance risk. The number of cases of resistance from the International Survey of Herbicide Resistant Weeds [4] ranked and grouped by the legacy HRAC mode-of-action (data accessed in January 2020). Z includes subcategories Z1, Z2, Z3, Z4. Other includes mode-of-actions with 2 or fewer cases: F2, F4, K2, I. The example active ingredients are examples of commonly used active ingredients in the New Zealand context.

Resistance Risk	HRAC Herbicide MoA Groups		Example active ingredient	Number of resistant ~~taxa~~ species worldwide	% of the worldwide total
High	B	ALS inhibitor	flumetsulam	165	32
	C1	PSII inhibitors (triazines)	atrazine	74	15
	A	ACCase inhibitors	pinoxaden	48	9
	G	EPSP synthase inhibitors	glyphosate	47	9
Medium	O	Synthetic auxin	MCPA	41	8
	D	PSI electron diverters	paraquat	32	6
	C2	PSII inhibitors (ureas and amides)	isoproturon	29	6
Low	E	PPO inhibitors	carfentrazone	13	3
	K1	Microtubule inhibitors	trifluralin	12	2
	N	Lipid inhibitors	triallate	10	2
	K3	Long-chain fatty acid inhibitors	flufenacet	7	1
	F3	Carotenoid biosynthesis (unknown target)	amitrole	6	1
	Z*	Anti-microtubule mitotic disrupter	flamprop	6	1
Very-low	C3	PSII inhibitors (nitriles)	ioxynil	4	<1
	F1	Carotenoid biosynthesis inhibitors	diflufenican	4	<1
	H	Glutamine synthase inhibitors	glufosinate	4	<1
	L	Cellulose inhibitors	dichlobenil	3	<1
	-	Other MoA	-	6	1

Herbicide use

Current herbicide use in wheat and barley fields involves 75 unique active ingredients in 16 mode-of-action groups (we show the most important herbicide groups in ~~Table 2~~Table 2~~3~~3). Barley and wheat have shared patterns of herbicide usage with respect to mode-of-action (~~Table 2~~Table 2~~3~~3) and active-ingredients. Synthetic auxins (HRAC group O) were represented in higher proportions than any other class of herbicide (a total usage rate of 26%). ALS-inhibitors (B), PDS-inhibitors (F1) and EPSPS-inhibitors (G) were highly used herbicide groups with >10% total usage, and acetyl coenzyme-A carboxylase inhibitors (A) and photosystem-II disrupters (C) were moderately used (total <10%). EPSPS-inhibitors (G) were used in larger proportions in barley (18%) ~~versus~~ compared to wheat (12%); ~~alternately~~conversely, farmers used K3 herbicides significantly more in wheat at 12% compared to barley at 4%. Glyphosate (MoA group G) is mostly used (>95%) used to control weeds pre-sowing of the cereal crops, for termination of the previous crop or pre-establishment weed control. It is very rarely used as crop pre-harvest desiccant.

~~Table 2~~Table 2~~3~~. **Ranked herbicide mode-of-action usage proportions.** The percentage of fields that received herbicide applications, grouped by HRAC MoA categories~~HRAC categories~~. Data are sourced from the ProductionWise® platform, entered by farmers. It represents a record of herbicide use from 5026 barley and 7647 wheat fields, collected during 2017 and 2018. The following 10 HRAC MoA groups each accounted for less than 1% of herbicide applications, N, F4, K1, I, H, F3, Z, L, F2 & K2.

Mode-of-action	Barley %	Wheat %	Total %
O	30	24	26
B	16	17	16
F1	14	17	16
G	18	12	14
K3	4	12	9
A	8	6	7
C1	3	5	4
C2	1	4	3
C3	2	1	1
E	2	<1	1
D	1	1	1

Individual herbicide usage ranking for each crop is displayed in Fig 1, ranked by the wheat herbicide use. The ten most used herbicides (in order) for barley were: glyphosate (HRAC group G), diflufenican (F1), fluroxypyr (O), MCPA (O), chlorsulfuron (B), pinoxaden (A), iodosulfuron (B), flufenacet (K3), mecoprop (O) and clopyralid (O). Flufenacet and terbuthylazine were the only high-use pre-emergent active-ingredient used in wheat (the latter can also be used post-emergent), both were used less in barley crops.

Fig 1. The ten most common herbicides applied in New Zealand wheat and barley fields. –

The ten most used herbicides as a percentage of total application instances documented by farmers in New Zealand wheat and barley fields, here ordered by observations in wheat crops. The herbicides that were not ranked in the top ten for each crop respectively were included here for both of the crops for completeness.

Cases of resistance by species and risk scoring

The documented cases of herbicide-resistance for medium and high-risk species and all herbicide mode-of-action combinations are shown in ~~Table S1~~Table 3~~1~~ (a total of 46~~5~~ medium and high-risk species). High-risk species were the ~~the nine~~eight grasses *Avena fatua, Avena sterilis, Digitaria*

sanguinalis, *Echinochloa crus-galli*, *Lolium multiflorum*, *Lolium perenne*, *Phalaris minor*, *Poa annua* and ~~the~~ eight~~nine~~ broadleaf weeds *Amaranthus powellii*, *Chenopodium album*, *Erigeron bonariensis*, *Erigeron sumatrensis*, *Raphanus raphanistrum*, *Senecio vulgaris*, *Solanum nigrum*, *Stellaria media*. Considering our list of weed species known in wheat and barley in New Zealand, we can see that the number of weed species with herbicide resistance documented worldwide varies across HRAC herbicide groups B (34 species), C1 (21), G (16), O (13) and A (12) (~~Table S1~~Table 3~~1~~). *Poa annua*, *Echinochloa crus-galli*, *Lolium* spp., *Erigeron sumatrensis*, *Raphanus raphanistrum* and *Avena fatua* have ~~many~~ twenty or more recorded cases ~~in~~ that occur in five or more unique mode-of-action groups each~~five or more unique mode-of-action each (>20)~~.

Table 3. Herbicide resistance risk scores and cases for herbicide mode-of-action groups and species. For weeds of wheat and barley in New Zealand we document the number of worldwide cases of herbicide resistance within different HRAC [26] groups. Data are sourced from the International Survey of Herbicide-Resistant Weeds [4] and concerns unique modes-of-action within reported cases of herbicide resistance. Species-risk and herbicide-risk are either low (1), medium (2), or high (3). Total cases and species per herbicide mode-of-action across worldwide cases [4] are presented at the bottom of the table. Symbols designate species with herbicide resistance cases detected in New Zealand wheat and barley *, or in other crops † [11,28]. Table footnotes report low-risk weeds.

**Species**	**Species Risk**	**A**	**B**	**C1**	**C2**	**C3**	**D**	**E**	**F1**	**F2**	**F3**	**F4**	**G**	**H**	**L**	**K3**	**K1**	**N**	**O**	**Z**	**Groups**	**Total Unique Cases**
**Herbicide Risk**		3	3	3	2	1	2	1	1	1	1	1	3	1	1	1	1	1	2	1		
Amaranthus powelii	3	0	8	8	0	0	0	0	0	0	0	0	0	0	0	0	0	0	0	0	2	16
*Avena fatua**	3	38	19	0	0	0	0	1	0	0	0	0	0	0	0	1	1	9	0	8	7	77
Avena sterilis	3	15	9	0	0	0	0	0	0	0	0	0	2	0	0	0	0	0	0	2	4	28
Brassica rapa	2	0	1	1	0	0	0	0	0	0	0	0	2	0	0	0	0	0	1	0	4	5
Bromus catharticus	2	0	0	0	0	0	0	0	0	0	0	0	1	0	0	0	0	0	0	0	1	1
Bromus diandrus	2	1	1	0	0	0	0	0	0	0	0	0	1	0	0	0	0	0	0	0	3	3
Bromus secalinus	2	0	2	0	0	0	0	0	0	0	0	0	0	0	0	0	0	0	0	0	1	2
Bromus sterilis	2	1	2	0	0	0	0	0	0	0	0	0	0	0	0	0	0	0	0	0	2	3
Capsella bursa-pastoris	2	0	6	2	0	0	0	0	0	0	0	0	0	0	0	0	0	0	0	0	2	8
Carduus nutans†	2	0	0	0	0	0	0	0	0	0	0	0	0	0	0	0	0	0	1	0	1	1
Chenopodium album†	3	0	7	41	0	0	0	0	0	0	0	0	0	0	0	0	0	0	1	0	3	49
Cirsium arvense	2	0	0	0	0	0	0	0	0	0	0	0	0	0	0	0	0	0	2	0	1	2
Critesion murinum	2	3	1	0	0	0	4	0	0	0	0	0	1	0	0	0	0	0	0	0	4	9

Convolvulus arvensis	2	0	0	0	0	0	1	0	0	0	0	0	0	0	0	0	0	0	0	0	1	1
Digitaria sanguinalis	3	7	3	3	0	0	0	0	0	0	0	0	0	0	0	0	0	0	0	0	3	13
Echinochloa crus-galli	3	14	16	6	11	0	0	0	0	0	0	1	1	0	2	3	0	2	7	0	10	63
Erigeron bonariensis	3	0	1	2	0	0	6	0	0	0	0	0	13	0	0	0	0	0	0	0	4	22
Erigeron sumatrensis	3	0	7	1	0	0	9	0	0	0	0	0	10	0	0	0	0	0	1	0	5	28
Fallopia convolvulus	2	0	2	2	0	0	0	0	0	0	0	0	0	0	0	0	0	0	0	0	2	4
Fumaria densiflora	2	0	0	0	0	0	0	0	0	0	0	0	1	0	0	0	1	0	0	0	2	2
Galium aparine	2	0	4	0	0	0	0	0	0	0	0	0	0	0	0	0	0	0	3	0	2	7
Lactuca serriola	2	0	5	0	0	0	0	0	0	0	0	0	1	0	0	0	0	0	1	0	3	7
Lamium amplexicaule	2	0	1	0	0	0	0	0	0	0	0	0	0	0	0	0	0	0	0	0	1	1
*Lolium multiflorum**	3	55	37	0	0	0	2	0	0	0	1	0	27	3	0	5	0	0	0	0	7	130
*Lolium perenne**	3	4	5	0	0	0	0	0	0	0	1	0	4	1	0	0	0	0	0	0	5	15
Persicaria maculosa†	2	0	1	4	0	0	0	0	0	0	0	0	0	0	0	0	0	0	0	0	2	5
Phalaris minor	3	9	4	0	0	0	0	0	0	0	0	0	0	0	0	0	0	0	0	0	2	13
Phalaris paradoxa	2	7	1	1	0	0	0	0	0	0	0	0	0	0	0	0	0	0	0	0	3	9
Plantago lanceolata	2	0	0	0	0	0	0	0	0	0	0	0	1	0	0	0	0	0	1	0	2	2
Poa annua†	3	3	10	19	0	0	2	0	0	0	1	0	7	0	0	0	7	1	4	0	9	54
Polygonum aviculare	2	0	0	1	0	0	0	0	0	0	1	0	0	0	0	0	0	0	0	0	2	2
Raphanus raphanistrum	3	0	9	1	0	0	0	0	3	0	0	0	1	0	0	0	0	0	6	0	5	20
Rumex acetosella	2	0	0	1	0	0	0	0	0	0	0	0	0	0	0	0	0	0	0	0	1	1

Rumex obtusifolius	2	0	1	0	0	0	0	0	0	0	0	0	0	0	0	0	0	0	0	0	1	1
Senecio vulgaris	3	0	2	13	0	1	0	0	0	0	0	0	0	0	0	0	0	0	0	0	3	16
Silene gallica	2	0	1	0	0	0	0	0	0	0	0	0	0	0	0	0	0	0	0	0	1	1
Solanum americanum†	2	0	0	0	0	0	2	0	0	0	0	0	0	0	0	0	0	0	0	0	1	2
Solanum nigrum†	3	0	0	11	0	0	3	0	0	0	0	0	0	0	0	0	0	0	0	0	2	14
Sonchus asper	2	0	5	1	0	0	0	0	0	0	0	0	0	0	0	0	0	0	0	0	2	6
Sonchus oleraceus	2	0	3	0	0	0	0	0	0	0	0	0	1	0	0	0	0	0	2	0	3	6
Spergula arvensis	2	0	1	0	0	0	0	0	0	0	0	0	0	0	0	0	0	0	0	0	1	1
Stellaria media†	3	0	20	1	0	0	0	0	0	0	0	0	0	0	0	0	0	0	2	0	3	23
Tripleurospermum inodorum	2	0	7	0	0	0	0	0	0	0	0	0	0	0	0	0	0	0	0	0	1	7
Urtica urens	2	0	0	1	0	0	0	0	0	0	0	0	0	0	0	0	0	0	0	0	1	1
Vicia sativa	2	0	1	0	0	0	0	0	0	0	0	0	0	0	0	0	0	0	0	0	1	1
Vulpia bromoides	2	0	0	2	0	0	1	0	0	0	0	0	0	0	0	0	0	0	0	0	2	3
**Total cases count**		157	203	122	11	1	30	1	3	0	4	1	74	4	2	9	9	12	32	10		683
**Total species count**		12	34	21	1	1	9	1	1	0	4	1	16	2	1	3	3	3	13	2		127

[a]We report the low risk weeds: *Achillea millefolium, Agrostis capillaris, Amaranthus deflexus, Amsinckia calycina, Aphanes arvensis, Arrhenatherum elatius, Avena barbata, Barbarea intermedia, Brassica napus, Bromus hordeaceus, Calandrinia compressa, Calandrinia menziesii, Cardamine flexuosa, Cerastium glomeratum, Chenopodiastrum murale, Cirsium vulgare, Crepis capillaris, Crepis setosa, Dactylis glomerata, Elytrigia repens, Erodium cicutarium, Erodium moschatum, Festuca rubra, Fumaria bastardii, Fumaria muralis, Fumaria officinalis, Gamochaeta coarctata, Gamochaeta purpurea, Geranium molle, Leontodon saxatilis, Lepidium didymum, Lotus pedunculatus, Lysimachia arvensis, Malva neglecta, Malva parviflora, Matricaria discoidea, Oxalis debilis, Oxalis latifolia, Phalaris aquatica, Phalaris canariensis, Ranunculus repens, Sherardia arvensis, Silene vulgaris, Sisymbrium officinale, Solanum sarrachoides, Stachys arvensis, Taraxacum officinale, Trifolium pratense, Trifolium repens, Veronica arvensis, Veronica persica, Vicia hirsuta, Vicia lathyroides, Viola arvensis, Vulpia myuros*

~~Cumulative~~ The cumulative risk scores ~~depicted~~ shown in ~~(~~Fig 2~~)~~ ~~weight species that have high "intrinsic" species risk scores, with cases in high-risk modes-of-action and show~~gives a higher score to weeds with resistance to multiple modes-of-action~~modes of action~~. ~~A large proportion of the cumulative species risk is explained by multiple cases within high-use modes-of-action. We see that some~~Because of this species with high overall risk scores may have ~~have fewer~~relatively few cases of resistance detected overall but their score is inflated by cases of resistance to multiple HRAC modes-of-action. For ~~, for~~ example *Lolium perenne* (15 cases in 5 modes-of-action) has a slightly lower risk score overall compared to its congener *Lolium multiflorum* (130 cases in 7 modes-of-action)~~ but significantly fewer cases~~. ~~In New Zealand at least, these species freely hybridize, making identification difficult. Species risk scores emphasize cases where resistance to multiple modes-of-action have been found.~~ *Chenopodium album* is an example of a relatively low scoring weed that has had ~~many~~ 49 cases of ~~documented~~ resistance documented but ~~in~~ within only ~~few~~ HRAC modes-of-action (Fig 2, Table 3~~1~~)~~; the risk is skewed towards herbicides in those groups~~. ~~This shows how the risk can be broad across groups or skewed toward certain groups.~~ Risks can be skewed toward certain herbicide modes-of-action, or species. Weeds with the ten highest cumulative scores are *Echinochloa crus-galli, Poa annua, Lolium multiflorum, Erigeron sumatrensis, Raphanus raphanistrum, Lolium perenne, Erigeron bonariensis, Avena fatua, Avena sterilis, Digitaria sanguinalis.* This shows that 7 out of 10 of the high risk species are grass-weeds. Grass-weeds are over-represented in global cases of resistance.

Fig 2. Number of cases ~~globally~~ of herbicide resistance (globally) by weed species and cumulative herbicide resistance risk scores. The Sum of Risk (cumulative risk) scores on the x-axis is the sum of the herbicide-risk × species-risk scores (from ~~Table S1~~Table 3~~1~~) for each HRAC herbicide mode-of-action group that had documented cases of resistance somewhere in the world. We also show the number of resistance cases documented in the world. The green dashed line shows the 10 cases needed for a species to be designated high risk (score 3). We distinguished the proportion of the risk and resistance cases that matched with the high-use HRAC mode-of-action groups (A, B, C1, F1, G, K3 & O) used by wheat and barley farmers in New Zealand (light-green for risk, and grey for cases).

Discussion

We present evidence about the propensity of individual weed species to develop resistance in a curated list (Table S1) of weed species known to occur in New Zealand wheat and barley fields [22]. The ten highest cumulative risk scores, in order *Echinochloa crus-galli, Poa annua, Lolium multiflorum, Erigeron sumatrensis, Raphanus raphanistrum, Lolium perenne, Erigeron bonariensis, Avena fatua, Avena sterilis and Digitaria sanguinalis*. ~~, and *Stellaria media*~~[11]~~, Species to species differences in distribution, abundance and phenology (e.g. germination timing) will mean that our risk scores do not capture field level differences in selection pressure from farmer herbicide applications in wheat in barley.~~ We~~We~~ expect to see ~~detect~~ novel~~ew~~ instances of resistance in common weeds with moderate-to-high risk scores (in order): *Erigeron* spp., *Raphanus raphanistrum, Chenopodium album, Senecio vulgaris, Phalaris* spp., *Bromus diandrus, Sonchus oleraceus,* ~~*Stellaria media*,~~ *Solanum nigrum* and *Persicaria maculosa*.~~*Chenopodium album, Brassica rapa, Senecio*~~

vulgaris, *Phalaris* spp., *Bromus diandrus*, and *Sonchus oleraceus* Species to species differences in distribution, abundance and phenology (e.g. germination timing) will mean that our risk scores do not capture field level differences in selection pressure from farmer herbicide applications in wheat in barley. As such, emerging grass weeds *Echinochloa crus-galli and Digitaria sanguinalis* are identified withas high-risk scores despite their currently limited distribution. . Emerging weeds *Echinochloa crus-galli*, *Digitaria sanguinalis* and *Raphanus raphanistrum* are not widespread but may beare identified ase *selected* as being hi high-risk weeds. emerging problems in wheat and barley fields in New Zealand. Species to species differences in distribution, abundance and phenology (e.g. germination timing) will mean that our risk scores do not capture field level differences in selection pressure from farmer herbicide applications in wheat in barley. . For example, in spring-sown cereals, C_4 grasses like *Echinochloa crus-galli* may often germinate after herbicides are applied, but when sowings get delayed because of wet spring weather then spraying will impact these seedlings. Farmer implementation of integrated weed management will vary at a fine spatial scale too, impacting selection pressure. Nevertheless, the information we gathered provides a useful indicator wheat and barley weeds with a known propensity to develop resistance. By being aware of all the weed species that are high-risk we shoulds improve detection of resistance cases in future. By acting on field experience herbicide-resistance researchers may be biased toward the most frequent survivors of weed management, and may not sample less abundant but high-risk species. Farmers should be informed about the high-risk species and herbicide combinations, so as to avoid high-risk behaviors, or at least keep an eye out for problems they will select for. The unique data we obtained about actual herbicide use (Table 2) will also help us to prioritize herbicides for testing of weeds that we will collect in future surveys designed to detect herbicide resistance.

Estimating the overall prevalence of herbicide resistance in all the major farming sectors in New Zealand could cost $1-3 million NZD depending on sampling rates [8]. An obvious concern is that detection rates for herbicide-resistant weeds will necessarily underestimate the true rate, given that surveyors may miss individual weed species, resistant plants, or seeds during farm visits [8] or they miss cases by screening for the wrong herbicides. Surveys have been initiated for the arable sector in New Zealand's Canterbury region where wheat and barley are important crops. They will sample about 20% of ca. 800 arable farms at an estimated cost of ca. $154,000. Given these high survey costs, it is important to take steps to improve detection rates, the high-risk species identified here should be targeted during surveys. Without this list we could be biased toward a smaller number of known problem weeds, such as *Lolium* spp. and *Avena fatua*. A review of the New Zealand literature shows only two reports of resistant species in wheat and barley [7,11]A review of the New Zealand literature shows only two reports of resistant species in wheat and barley [7,11]. To date, there has been haphazard detection of herbicide resistance and variable levels of scientific investigation [7,11][7,8]. As are result, there are gaps in our knowledge about resistance in New Zealand about the prevalence [11][8] and biochemical and genetic mechanisms [7]. Only a few cases of resistance were documented in wheat and barley in New Zealand, This contrasts with the high numbers of resistances cases seen worldwidedespite worldwide instances of weed resistance in for these crops these crops,(-77 cases in wheat, and 30 cases in barley) [4]. One might expect cases to be reported quickly, given that selection for rare mutations that confer resistance is infrequent but instantaneous. But surviving individuals and progeny may take a few years to increase to detectable levels (in a field) under continuous selection pressure [30]. Via ad-hoc reporting,In New Zealand resistance was detected in 2014 for *Avena fatua*

to acetyl coenzyme-A carboxylase (group A, ACCase)[31], and in 2017 for ryegrasses (*Lolium perenne* and *L. multiflorum*) to ACCase and acetolactate synthase targeting herbicides (ALS herbicide, group B) [31,32]. – Another earlierA –1996 report of *Stellaria media* resistance to chlorsulfuron and tribenuron (group B) in an oat crop may indicate elevated risk given the shared agronomic practices shared between these cereal crops [33]. ResistantThese resistant weeds previously detected in New Zealand cereals *Lolium* spp., and *Avena* spp. and *Stellaria media*, are therefore likely to continue showing upto be observedas resistance cases in wheat and barley. It seems likely that farmers are under-reporting resistance cases perhaps because alternative weed control measures can keep problems manageable. The recency of the reports of resistance in New Zealand ryegrass species is a little surprising, when contrasted with Australian cases of *Lolium rigidum* herbicide resistance (in dryland wheat) which were detected within three years of introducing a new herbicide; and models suggest that under standard herbicide application scenarios, resistant allele frequencies will pass 50% within 10 years [31]. Ostensibly, selection of rare mutations that confer resistance is infrequent but instantaneous, with surviving individuals and progeny increasing to detectable levels (in a field) within a few years under continuous selection pressure. The best explanation of rare detections in this high-risk crop type in New Zealand is that on-farm cases of resistance are rarely reported to weed scientists. Even Evenwhen they are reported in the literature it could be years or decades after the problem was, or should have been, noticed. Clearly littleIn the absence of a systematic approach, little is currently known about the spatial and temporal patterns in the development of herbicide resistance development in New Zealand.

Ostensibly, selection of rare mutations that confer resistance is infrequent but instantaneous, with surviving individuals and progeny increasing to detectable levels (in a field) within a few years under continuous selection pressure[32]. The best explanation of rare detections in this high-risk crop type in New Zealand is that on-farm cases of resistance are rarely reported to weed scientists. Even Evenwhen they are reported in the literature it could be years or decades after the problem was, or should have been, noticed. Clearly little is known about spatial and temporal patterns in the development of herbicide resistance in New Zealand. A review of the New Zealand literature shows only two reports of resistant species in wheat and barley [7,11][7,8]. At least 12 out of 48 randomly selected arable farms on the South Island of New Zealand had herbicide-resistant weeds in a herbicide resistance survey we initiated in 2019 (unpublished resistance survey data). These data indicate that we are underestimating the amount of herbicide resistance that is occurring in arable farming systems.

Estimating the overall prevalence of herbicide resistance in all the major farming sectors in New Zealand could cost $1-3 million NZD depending on sampling rates [11][8]. An obvious concern is that detection rates for herbicide-resistant weeds will necessarily underestimate the true rate, given that surveyors may miss individual weed species, resistant plants, or seeds during farm visits [11][8] or they could miss cases by screening for the wrong herbicides. Surveys have been initiated for the arable sector in New Zealand's Canterbury region where wheat and barley are important crops. They will sample about 20% of ca. 800 arable farms at an estimated cost of ca. $154,000. Given these high survey costs, it is important to take steps to improve detection rates, the high-risk species identified here should be targeted during surveys. Without this list we could be biased toward a smaller number of known problem weeds, such as *Lolium* spp. and *Avena fatua*.

~~We present evidence about the propensity of individual weed species to develop resistance in a curated list (Table S1) of weed species known to occur in New Zealand wheat and barley fields~~ [22]~~[19]. The ten highest cumulative risk scores, in order *Echinochloa crus-galli, Poa annua, Lolium multiflorum, Erigeron sumatrensis, Raphanus raphanistrum, Lolium perenne, Erigeron bonariensis, Avena fatua, Avena sterilis and Digitaria sanguinalis*. With respect to species risk, our results suggest that herbicide resistance in *Lolium* spp. and *Avena* spp. are likely to continue showing up as resistance cases in New Zealand wheat and barley. We expect to detect new cases in common weeds: *Stellaria media, Chenopodium album, Brassica rapa, Senecio vulgaris, Phalaris* spp., *Bromus diandrus*, and *Sonchus oleraceus*. *Echinochloa crus-galli, Erigeron sumatrensis, Raphanus raphanistrum, Erigeron bonariensis, Avena sterilis, and Digitaria sanguinalis* are not widespread but may be emerging problems in wheat and barley fields in New Zealand. Species to species differences in distribution, abundance and phenology (e.g. germination timing) will mean that our risk scores do not capture field level differences in selection pressure from farmer herbicide applications in wheat in barley. For example, in spring sown cereals, C_4 grasses like *Echinochloa crus-galli* may often germinate after herbicides are applied, but when sowings get delayed because of wet spring weather then spraying will impact these seedlings. Farmer implementation of integrated weed management will vary at a fine spatial scale too, impacting selection pressure. Nevertheless, the information we gathered provides a useful indicator wheat and barley weeds with a known propensity to develop resistance. Our weed risk ranks could improve detection of resistance cases in further surveys we plan to carry out for potentially herbicide-resistant weeds. By acting on field experience herbicide-resistance researchers may be biased toward the most frequent survivors of weed management, and may not sample less abundant but high-risk species. Farmers should be informed about the high-risk species and herbicide combinations, so as to avoid high risk behaviors, or at least keep an eye out for problems they will select for. The unique data we obtained about actual herbicide use (Table 2) will also help us to prioritize herbicides for testing of weeds that we will collect in future surveys designed to detect herbicide resistance.~~ The Moss protocol relies heavily on a herbicide-risk score (rank low, medium and high). We deviated from their approach slightly. This was necessary in part because the number of cases for different herbicide groups has increased since the Moss article was published. ~~The herbicide-risk is not necessarily a function of the number cases alone. Risk assessments should take into account the volumes used, years of product use, spatial extent of the applications, and the number of weeds treated. Estimations of these other factors can be made, but are likely to be inaccurate.~~ A case in point is our decision to include glyphosate as a high-risk herbicide. As recently as 2006 glyphosate was ranked as amongst the least likely to select for resistance [29], and is recognized as medium-risk by Moss. We ranked glyphosate (group G; ~~Table 1~~Table 1~~2~~) as high risk because worldwide the number of species showing resistance has increased to 47 cases, similar to group A (48 cases) which is universally regarded as high-risk. Group A herbicides were ranked as high-risk in the Moss protocol. Two cases of glyphosate resistance are known from New Zealand [7,11]~~[7,10][7,8]~~. If we had used the 10% threshold groups, A and G would be medium risk and species scores would have changed, but the top five ranked species would have remained the same ~~(both A and G were >9%)~~. We are aware that herbicide-risk is not just a function of the number associated cases of resistance. More complete risk assessments would ideally factor in the herbicide volumes used, years of product use, spatial extent and number of the applications, as well as the abundance of high-risk weeds in the areas treated.~~Other herbicide risk assessments used different~~

~~criteria. The issue with our, and Moss et al's herbicide-risk is that it does not factor in the number of years a product has been in use or the land area it has been applied to. A different risk assessment by Beckie ranked HRAC groups with A and B as high risk, C1 as moderate-high with G and O at low, based on expected number of herbicide applications thought to produce target-site resistance~~ [33]~~[31]~~ ~~Using Beckie's as our herbicide-risk ranking would produce different risk-scores.~~

Herbicide usage data in New Zealand have rarely been quantified, and only roughly via indirect sales data numbers and expert elicitation [34,35]~~[35,36]~~. The ProductionWise® system is used to record the on-farm use of chemical inputs and other information. This system (and similar tools) are valuable for ensuring farmer compliance with record-keeping regulations, supporting farmer decision-making, and guiding herbicide resistance prevention efforts. It also serves to capture industry-wide behaviour regarding agrichemical use which is how we have used it in this case. Herbicide resistance risk is ~~also~~ influenced by the other crops in the rotation, and temporal and spatial differences in weed composition. The relatively low rates of herbicide resistance detected in the New Zealand arable sector may ~~come as a benefit~~be a consequence of mixed-~~-~~crop –rotation systems. It is not uncommon to include a 1-3 year pasture rotation, and a complex crop sequence, for example, winter wheat, spring-sown peas or linseed, winter wheat or barley, followed by grass, and oilseed rape and back to winter wheat –~~It was thought that resistance may be rare because crop rotations are a mitigating factor for herbicide resistance risk in New Zealand. It is not uncommon to include a 1-3 year pasture rotation, and a crop sequence might go as follows: winter wheat, spring sown peas or linseed, winter wheat or barley, followed by ryegrass, and oilseed and back to winter wheat~~ [36]~~[37]~~. Cases of herbicide resistance we observe now only partly reflect current herbicide use and may, in fact, implicate historic selection by herbicides that have fallen out of favour. We think advanced record-keeping tools like this and related decision support systems have real potential to improve outcomes for farmers and scientists.

Within New Zealand wheat and barley fields herbicide use and risk of resistance are greatest within HRAC groups (in order) O, B, G, A and C1. Surprisingly, there have been few cases of resistance to ALS-inhibitors (B) and synthetic auxins (O) documented to date given that they have been the most commonly applied herbicides for more than 20 years [37,38]. Field observations show that broadleaf weeds are rarer in the wheat fields prior to harvest compared to grass weeds, but survivors should be tested for herbicide resistance. Group B herbicides have been implicated in a large number of resistance cases in both broadleaf and grass weeds, including 34 species known to occur in wheat and barley in NZ (Table 3~~1~~). Groups F1 and K3 have a relatively low risk of developing resistance based on historical occurrences even though they are highly used in New Zealand.

Examining our herbicide use information in the context of published herbicide evolution models can provide important insights. Models based on dryland wheat systems and the weed *Lolium rigidum* [39] showed that resistance rate evolution was not slowed by simple herbicide rotations (i.e. annual with few herbicides). Importantly the use of soil-applied herbicides, particularly trifluralin (group K1), full-rate mixes of herbicides, and complex 8-year long rotations were shown to delay resistance evolution by years, and in some scenarios by decades [39]. There is a chance that resistance cases in

soil-applied pre-emergent herbicides are under-reported compared to post-emergent ones (they are harder to test). For now, resistance evolution in those herbicides appears to be slower. Trifluralin did not feature amongst the most common herbicides in the farmer herbicide use data we obtained for 2017 and 2018 (<0.5% of field applications); the only frequently used pre-emergent herbicides were flufenacet and terbuthylazine. ~~.~~Farmers should be informed about the high-risk species and herbicide combinations, so as to avoid high-risk behaviors, or at least keep an eye out for problems they will select for. Farmer decision support platforms~~, ~~ and research and extension efforts in New Zealand should emphasize mechanical and cultural control measures, the use of soil-applied pre-emergent herbicides, full-strength label-rate herbicide mixtures, crop rotation to utilize herbicides otherwise unavailable and herbicide rotations of key active ingredients to achieve maximum control and to reduce the rate at which herbicide resistance evolves in weed populations.

Conclusions

A European protocol [20]~~[19][17]~~ ~~–~~, designed primarily to assist in herbicide authorization procedures, was adapted to assess the risk of herbicide resistance evolving in 100 different weed species known to occur in New Zealand wheat and barley crops. More than half the weeds~~designed for a herbicide authorization process was adapted to assess the risk that different weed species known in New Zealand wheat and barley could become resistant. More than half the weeds (55) we assessed were low-risk,~~ 30~~29 were medium-risk and 16 high-risk, the top ten were~~ weeds (55) we assessed were low-risk, 29 were medium-risk and 16 high-risk. The 10 species posing the highest resistance risk were: *Echinochloa crus-galli*, *Poa annua*, *Lolium multiflorum*, *Erigeron sumatrensis*, *Raphanus raphanistrum*, *Lolium perenne*, *Erigeron bonariensis*, *Avena fatua*, *Avena sterilis* and *Digitaria sanguinalis*. To provide important context we also report on herbicide use patterns in New Zealand wheat and barley fields. We are planning extensive surveys in New Zealand to detect new cases of herbicide resistance. The risk assessment outlined in this paper will enable us to prioritise those weeds identified as posing a high resistance risk and, consequently, make better use of available resources. The risk assessment procedure as described in this paper has the potential to be a useful tool for evaluating the risk of herbicide resistance in a wide range of different weed species in other countries too.

Acknowledgments

Ian Heap's International survey of herbicide resistant weeds was an important resource for this article. This work was supported by the Ministry of Business, Innovation and Employment [grant number C10X1806] to AgResearch Ltd.: "Improved weed control and vegetation management to minimize future herbicide resistance." Shona Lamoureaux, Phil Hulme, Trevor James, Hossein Ghanizadeh, Ian Heap and Graeme Bourdôt provided helpful comments on a draft.

References

1. Oerke E-C. Crop losses to pests. J Agric Sci. 2006;144: 31–43.

2. Stephenson GR. Pesticide Use and World Food Production: Risks and Benefits. Expert Committee on Weeds - Comité d'experts en malherbologie. Banff, Canada: Canadian Weed Science Society; 2000. pp. 9–15. doi:10.1021/bk-2003-0853.ch015

3. Thorne ME, Young FL, Pan WL, Alldredge JR. No-till spring cereal cropping systems reduce wind erosion susceptibility in the wheat/fallow region of the Pacific Northwest. J Soil Water Conserv. 2003; 9.

4. Heap I. International survey of herbicide resistant weeds - weedscience.org. 2020 [cited 25 Jan 2020]. Available: http://www.weedscience.org/

5. Baucom RS. The remarkable repeated evolution of herbicide resistance. Am J Bot. 2016;103: 181–183. doi:10.3732/ajb.1500510

6. Powles SB, Yu Q. Evolution in Action: Plants Resistant to Herbicides. Annu Rev Plant Biol. 2010;61: 317–347. doi:10.1146/annurev-arplant-042809-112119

7. Ghanizadeh H, Harrington KC. Herbicide resistant weeds in New Zealand: state of knowledge. N Z J Agric Res. 2019; 1–12. doi:10.1080/00288233.2019.1705863

8. Millner JP, Roskruge NR. The New Zealand arable industry. In: Dymond JR, editor. Ecosystem services in New Zealand – conditions and trends. Manaaki Whenua Press, Landcare Research; 2013. pp. 102–114.

9. Watson E. The highest wheat yield. In: Guinness World Records [Internet]. 17 Feb 2017 [cited 13 May 2020]. Available: https://www.guinnessworldrecords.com/world-records/highest-wheat-yield

10. Darling W. The highest barley yield. In: Guinness World Records [Internet]. 23 Jan 2015 [cited 13 May 2020]. Available: https://www.guinnessworldrecords.com/world-records/68919-highest-barley-yield

11. Buddenhagen CE, Gunnarsson M, Rolston P, Chynoweth RJ, Bourdot G, James TK. Costs and risks associated with surveying the extent of herbicide resistance in New Zealand. N Z J Agric Res. 2019;0: 1–19. doi:10.1080/00288233.2019.1636829

12. Broster JC, Koetz EA, Wu H. Herbicide resistance frequencies in ryegrass (*Lolium* spp.) and other grass species in Tasmania. Plant Prot Q. 2012;27: 36–42.

13. Broster JC, Koetz EA, Wu H. Herbicide resistance levels in annual ryegrass (*Lolium rigidum* Gaud.) in southern New South Wales. Plant Prot Q. 2011;26: 22–28.

14. Broster JC, Koetz EA, Wu H. A survey of southern New South Wales to determine the level of herbicide resistance in brome grass and barley grass populations. In: Zydenbos SM, editor. Seventeenth Australasian Weeds Conference. Christchurch, New Zealand; 2010. pp. 274–277.

15. Owen MJ, Goggin DE, Powles SB. Non-target-site-based resistance to ALS-inhibiting herbicides in six *Bromus rigidus* populations from Western Australian cropping fields. Pest Manag Sci. 2012;68: 1077–1082. doi:10.1002/ps.3270

16. Owen MJ, Martinez NJ, Powles SB. Herbicide resistance in *Bromus* and *Hordeum* spp. in the Western Australian grain belt. Crop Pasture Sci. 2015;66: 466. doi:10.1071/CP14293

17. Owen MJ, Martinez NJ, Powles SB. Multiple herbicide-resistant wild radish (*Raphanus raphanistrum*) populations dominate Western Australian cropping fields. Crop Pasture Sci. 2015;66: 1079. doi:10.1071/CP15063

18. Menchari Y, Camilleri C, Michel S, Brunel D, Dessaint F, Le Corre V, et al. Weed response to herbicides: regional-scale distribution of herbicide resistance alleles in the grass weed *Alopecurus myosuroides*. New Phytol. 2006;171: 861–874. doi:10.1111/j.1469-8137.2006.01788.x

19. Beckie HJ, Thomas AG, Stevenson FC. Survey of herbicide-resistant wild oat (*Avena fatua*) in two townships in Saskatchewan. Can J Plant Sci. 2002;82: 463–471. doi:10.4141/P01-067

20. Moss S, Ulber L, den Hoed I. A herbicide resistance risk matrix. Crop Prot. 2019;115: 13–19. doi:10.1016/j.cropro.2018.09.005

21. PP 1/213 (4) Resistance risk analysis. EPPO Bull. 2015;45: 371–387. doi:10.1111/epp.12246

22. Bourdôt GW, Hurrell GA, Saville DJ. Weed flora of cereal crops in Canterbury, New Zealand. N Z J Crop Hortic Sci. 1998;26: 233–247. doi:10.1080/01140671.1998.9514059

23. Rolston MP, Archie WJ, Reddy K, Dastgheib F. Grass weed control and herbicide tolerance in cereals. N Z Plant Prot. 2003;56: 220–226. doi:10.30843/nzpp.2003.56.6095

24. Kon KF, Follas GB, James DE. Seed dormancy and germination phenology of grass weeds and implications for their control in cereals. N Z Plant Prot. 2007;60: 174–182. doi:10.30843/nzpp.2007.60.4597

25. Breitwieser I, Brownsey PJ, Heenan PB, Nelson WA, Wilton AD. Flora of New Zealand Online. 2010 [cited 15 Feb 2020]. Available: http://www.nzflora.info/

26. HRAC. Global Classification Lookup. In: Herbicide Resistance Action Committee [Internet]. 2020 [cited 20 Jan 2020]. Available: https://hracglobal.com/tools/classification-lookup

27. ProductionWise – ProductionWise by Grain Growers. 2020 [cited 25 Feb 2020]. Available: https://productionwise.co.nz/

28. R Core Team. R: A Language and Environment for Statistical Computing. Vienna, Austria: R Foundation for Statistical Computing; 2020. Available: https://www.R-project.org/

29. Wickham H. ggplot2: Elegant Graphics for Data Analysis. Springer-Verlag New York; 2016. Available: http://ggplot2.org

30. Jasieniuk M, Brûlé-Babel AL, Morrison IN. The Evolution and Genetics of Herbicide Resistance in Weeds. Weed Sci. 1996;44: 176–193. doi:10.1017/S0043174500093747

31. Harrington KC, Chynoweth RJ. Have wild oats in Canterbury become resistant to herbicides. N Z Plant Prot. 2014;67: 331–331. doi:10.30843/nzpp.2014.67.5778

32. Gunnarsson M, James TK, Chynoweth RJ, Rolston MP. An evaluation of the resistance of annual and perennial ryegrass to herbicides. N Z Plant Prot. 2017;70: 165–170.

33. Bourdôt GW. The status of herbicide resistance in New Zealand weeds. Pestic Resist Prev Manag N Z Plant Prot Soc Rotorua N Z. 1996; 81–89.

34. NIWA Library catalogue › Details for: Patterns of pesticide use in New Zealand. Part 2, South Island 1986-1989 /. [cited 7 Jan 2020]. Available: https://library.niwa.co.nz/cgi-bin/koha/opac-detail.pl?resultid=2&biblionumber=272431&query_desc=

35. New Zealand, ACVM Group. ACVM registration information requirements for provisional registrations. Wellington, N.Z.: Ministry of Agriculture and Forestry; 1999.

36. Hampton JG, Rolston MP, Pyke NB, Green W, Lincoln A. Ensuring the long term viability of the New Zealand seed industry. Agron N Z. 2012; 12.

37. Holland PT, Rahman A. Review of trends in agricultural pesticide use in New Zealand. Ministry of Agriculture and Forestry Wellington,, New Zealand; 1999.

38. Manktelow D, Stevens P, Walker J, Gurnsey S, Park N, Zabkiewicz J, et al. Trends in pesticide use in New Zealand: 2004. Rep Minist Environ Proj No SMF4193. 2005.

39. Busi R, Powles SB, Beckie HJ, Renton M. Rotations and mixtures of soil- applied herbicides delay resistance. Pest Manag Sci. 2019; ps.5534. doi:10.1002/ps.5534

~~**Table S1. Herbicide resistance risk for herbicide mode-of-action groups and species.** For weeds of wheat and barley in New Zealand we document the number of worldwide cases of herbicide~~

~~resistance within different HRAC groups. Data are sourced from the International Survey of Herbicide-Resistant Weeds and concerns unique modes-of-action within reported cases of herbicide resistance. Species-risk and herbicide-risk are either low (1), medium (2), or high (3). Total cases and taxa per herbicide mode-of-action across worldwide cases are presented at the bottom of the table. Table footnotes report low-risk weeds.[a]~~

~~Table S2~~Table S1. The full list of weed species considered in our risk assessment for herbicide resistance in New Zealand Wheat and Barley crops. The list is derived from Bourdôt et al. [22]~~[21]~~~~[19]~~, with additions from literature [23,24]~~[22,23]~~~~[21,22]~~, expert knowledge and field observations made in January (late summer) of 2019 and 2020. Common name (in New Zealand) and family name are indicated. Nomenclature follows the New Zealand Flora [25]~~[24]~~~~[23]~~.

Reviewer #1:

Because frequency and abundance of weed species in the region have not been used in the scoring, the paper provides a picture of risks due to the current use of herbicides in the region, which is informative for the farmer about the intrinsic risk of an abundant species in its own farm. However, although I agree with intrinsic high risk species as stated on line 280-285, it is pity that the overall risk for the region is not presented.

> ***The regional risk is assessed! We use a published account of weeds, as well as expert knowledge to identify problem weeds in wheat and barley. The risk assessment considers the propensity of those weeds to develop resistance. It uses the most important factors, the crop specific region wide weed flora and region wide data about herbicide use. The selection pressure comes from the herbicides used and so captures the most important dimensions of the regional risk.***

L50-54 There is also an important survey on blackgrass in UK (see Hicks et al Nature Ecology & Evolution 2(3) 2018, DOI: 10.1038/s41559-018-0470-1). Probably other reports are available on surveys developed by national weed research associations in Spain, France (Columa), Italy, etc., including companies, extension service and farmers, and published in local conferences.

> ***That is another example of the "focus on one or two problematic species in a given crop" issue we highlight, and therefore does not need to be added.***

L142 It can be assumed that weed biology is already included in the inherent species score, but abundance is a key parameter. Abundance includes effects of farming systems: if many individuals still remain in the field, then the risk of developing resistance is higher than if the population is small. A score modifier to account for variation of abundance (e.g. 1 for low density and 3 for more than a million plant/ha, or another scale) could be used. Another factor is the species frequency in the studied region, which doesn't seem to be taken into account (is the Production Wise platform collecting main troublesome weed species?).

> ***While that is true, regional abundance and density data do not exist to be able to implement that. We acknowledge that distribution, abundance and phenology are important #(L272). ProductionWise does not gather any information on weeds. Because we identified high-risk species and herbicides the data are useful as a regional assessment. Farmers and experts can now interpret the observed weed abundance with an eye to the likely risk of resistance.***

Fig 2 Rather the low and high use HRAC groups I expected to see also the number of cases in New Zealand. I'm not clear with the low versus high use HRAC groups.

> ***We emphasize high use herbicides in our studied regions, because that selection pressure drives the evolution of resistance. We mention in the introduction that in***

New Zealand, there are only 12 instances of herbicide resistance in arable crops #(L41-43) and in the discussion delve further into New Zealand HR weeds (L238-244). The high and low usage herbicide groups come from ProductionWise data, which can be found on Table 2. The basis of deciding which groups are 'high use in New Zealand' is on L106-108. Cases of resistance have been indicated on Table 3.

Reviewer #2 Dr Stephen Moss:

Abstract: Generally fine but as likely to be most-read part I suggest a few minor changes. Do you need to say 'wheat and barley' or would 'cereal' suffice? Ditto in title.

We think wheat and barley is a more accurate statement for our case since the herbicide data focuses on that. Wheat and barley is more widely grown than oats, and depending on the reader, corn would also be considered to be a cereal…

I suggest:

15-18 'We estimated the risk of selecting for herbicide resistance in 100 weed species known to occur in wheat and barley crops on farms in New Zealand. A protocol was used that accounts for both the risk that different herbicides will select for resistance and each weed's propensity to develop herbicide resistance based on the number of cases worldwide.' ***Done***

24 'ALS-inhibitors were assessed as posing the greatest risk for more species than any other mode-of-action. ***Done***

25 I prefer 'Pre-emergence' but that may depend on journal style. ***Done***

26 '……. in this class commonly used by…..' Is this better than 'favoured'? ***Done***

Somewhere I think it would be worth stressing in abstract that 7 out of the high-risk 10 are grass-weeds. If space is limiting, I think the last sentence of abstract could go or 'farmer extension efforts' incorporated into previous sentence. ***Done***

Introduction: Good – acceptably concise and relevant. I suggest including some brief (one or two sentences) information on arable cropping in NZ and what proportion of that is accounted for by wheat and barley. Or at least to show that wheat and barley comprise a significant proportion of arable crops – you might even wish to mention that NZ currently holds world record for both barley and wheat yield/ha (I think that is correct). I would! ***Done***

32 Suggest: '……potential losses….' Losses of 23% don't actually commonly occur. ***Done***

41 Clarify 'cases'. To non-specialist reader it is vague and could mean number of fields or farms – although 'specialists' know why that term is appropriate. I would suggest stating number of weed species instead which is 13 according to Heap website country info. That lists 19 'cases' (29 April) although that duplicates some species. If it is 25 cases now (according to your reference), perhaps someone in NZ should provide Ian Heap with updated info. This is important if this paper is published. Likewise amend '12' in line 42 if appropriate. ***Done reworded to reflect species numbers in New Zealand, and documented instances of resistance, to distinguish from the usage of cases in the Heap database.***

45 'haphazard' is a bit unfair, although this is a valid point. I would dispute that my sampling and testing over 30 years has been 'haphazard'! Suggest 'unsystematic' as a better word – I would agree with that. End sentence after 'globally'. Then 'It reflects…..' ***Done***

50 comma needed after [9-14]. These 6 refs are all valid, but do you need them all? ***They are valid, focus on systematic efforts to detect any and all weed resistant cases. Also, they could be hard to find for other researchers working on a similar project. Leaving as is.***

53-54 Good points – one issue is that if resistance is perceived to be rare then hard to justify cost. If very widespread then why bother to do a survey? Money could be better spent. Also lack of infrastructure and personnel to conduct surveys may be as important as cost. Also, do surveys have much long-term value? Is a 20-year old survey of much value now – perhaps only as a benchmark? Perhaps money better spent on more 'durable' studies? Not saying that you need to consider these aspects here – perhaps in discussion as risk assessment relevant to survey priorities. ***Good points thanks for the insightful commentary.***

59 Suggest: '…….and their prior record of resistance in cereal fields elsewhere in the world.' ***We specifically looked at wheat and barley. It's an important distinction.***

62-64 This is fine and a good succinct sentence, but I wonder if it is worth emphasising more that risk assessment includes not only herbicide risk but also weed species risk for non-specialist readers, as this is not particularly intuitive? So, could read: 'They present a quantitative risk matrix using both herbicide-risk (some herbicides pose a higher risk than others) and species-risk (some weed species are more resistance-prone than others), with an optional score modifier designed to account for agronomic management practices that may reduce the risk.' ***Done***

67 Change: 'Individual field and farm scale risk is not assessed as this requires detailed information on past herbicide use, including timing etc…..' (This covers what has been applied - surely most important factor?) ***We changed the paragraph to emphasize importance of our herbicide application data set: ". This risk***

assessment is on an industry-wide scale informed by anonymized herbicide application data from wheat and barley fields. Risks were not assessed at the scale of individual farms and fields, this requires detailed information about herbicide timing, mixtures and rotations, and their interactions with weed biology, crop rotations and other cultural practices. All the high-risk weeds identified here should be targeted in surveys designed to detect herbicide-resistant weeds.

Materials and methods: Good – makes it clear what was done and why slightly different approaches to published protocol were adopted

76 Suggest: 'Most grasses and some …..' The wonders of Google mean that I can see, within 60 seconds, that two grass weed species were identified at species level in the paper cited….. ***Done***

83 Suggest: '….legacy herbicide mode of action (MoA) groups……' ***Done***

88-89 Suggest: '….legacy HRAC MoA group [24], with risk scores of 1, 2 or 3 given for low, medium or high risk respectively.' ***Done***

96 Suggest: '…..scored as '1'.' ('One' is a bit ambiguous). ***Done***

98 Suggest: 'Most recent….' ***Done but, "The most recent…"***

108-109 This is slightly confusing: 'The most used herbicides for each crop were characterized by weighting active ingredient amounts and the number of fields they were applied to.' It almost implies total a.i. weight was used. Not sure this sentence is needed. **Deleted the sentence.**

110 Why taxon? Species better? ***Changed to species.***

112 Suggest: '…..HRAC MoA group……' And I suggest elsewhere throughout paper. ***Done***

113 Suggest: '….herbicide type…. ***Done***

114 Suggest: '……the global number of resistance cases….' ***Done***

115 Suggest: 'To obtain the 'high', 'medium' and 'low' risk scores as used in the Moss protocol [17],…..' ***Done***

117-118 This sentence is slightly confusing and maybe could be improved. Is this correct? 'We assessed overall species-risk as the sum of the herbicide-risk multiplied by the "inherent" species-risk [17] combined for all relevant HRAC MoA herbicide groups, but only…….' (I think 'once' is confusing). The example you give below is useful. ***Done***

120 Suggest: '……weed species….' ***Done***

135 Suggest: '……to determine species-risk scores based on the number of cases of resistance worldwide, but we think this……' ***Done***

137 Suggest: ' ….45 'high' and 'medium' resistance risk species, many more than Moss et al……….' ***Done***

139 Suggest: 'The Moss protocol also used score modifiers that take into account resistance management practices including the use of non-chemical control measures' . ***Done***

139-142 Suggest: 'We did not use the score modifiers since these vary from field to field and our objective was slightly different. We acknowledge that actual risk of resistance development is determined mostly by the frequency and type of herbicide applied (selection pressure) interacting with characteristics of weed biology, distribution and abundance [5].' ***Done***

Results: Generally good and concise. One key paragraph needs improvement to improve clarity

145 Suggest: 'An additional 31 species were added…..' ***Done***

147 Suggest: ' ….resulting in a total of 100 weed species for consideration.' ***Done***

155 Suggest: Suggest: 'This resembles the table of Moss et al., [17] with…' ***Done***

Table 1 Use of 'taxa' is odd. Why not 'species' which is what is used on Heap's website? Taxa is a rather more general term and I cannot see any justification for use here. Would it also be useful to make the herbicide example the most commonly used ai for the group in NZ? Or if this has been done, state in title. ***Done***

171-172 Clarify if glyphosate used in-crop (for desiccation) or pre-sowing or both. Presumably both, but some comment about balance of use would be useful if only to stress that glyphosate use is very different to selective herbicides – non-specialists might assume use is primarily in GM crops, as in some other countries. Either here or earlier in M&M. ***Done "Glyphosate (MoA group G) is mostly used (>95%) used pre-sowing of the cereal crops, for termination of the previous crop or pre-establishment weed control. It is very rarely used as crop pre-harvest desiccant."***

Suggest: ' …….in barley (18%) compared with wheat (12%); conversely, farmers………' ***Done***

174 Again, 'HRAC MoA categories' ***Done***

Table 2 Suggest last 10 categories are simply summarised to save space and some statement added within table. e.g. 'The following 10 HRAC MoA groups each accounted for less than 1% of herbicide applications, N, F4, K1, I, H, F3, Z, L, F2 & K2.' ***Done***

181 I don't really see the point of stating actual % for barley when you haven't for wheat. Why not simply add % values to each of the bars in Fig 1. You could then say a bit about relative use of some individual herbicides in wheat and barley. Somewhere you should give some indication of relative area sown with wheat and

barley – in introduction or M&M. ***We added the percent values to the graph and clarified a comment about relative use under Table 2. The areas sown for wheat and barley are now in the introduction.***

183 Suggest: 'Flufenacet and terbuthylazine were the only herbicides used on a significant area pre-emergence (the latter can also be used post-emergence), both used less in barley crops.'

185 Suggest: '**Fig 1. The ten most commonly applied herbicides in New Zealand wheat and barley fields**.' ***Done***

190 Why taxon? Surely 'species' is better? ***Done***

193-194 Say eight grasses and eight broad-leafed weeds. ***Done***

201 Suggest: '….five or more unique mode-of-action groups each' What does>20 mean? Clarify. ***Done***

202-215 This paragraph covers the key output of this paper but is confusing and really needs a thorough re-write. Expand if necessary, to clarify results. Fig 2 contains a lot of information. I did wonder how much the 'cases' adds to this but I can see that it is relevant. Some definition of 'cases' is needed, as mentioned earlier. This is tricky as not easy to explain in a few words, but if this is explained in the M&M then no need to explain again, except perhaps to say refer to M&M. Should be made clear in Figure that cases is worldwide, as might be interpreted by casual reader as in NZ. ***Done Figure 2 clearly mentions global cases. Also added the following to the methods: "Cases are defined by the International Survey of Herbicide Resistant Weeds as unique combinations of weed species and HRAC herbicide mode-of-action (species x site of action)."***

212-215 I would make the point that 7 out of 10 are grass-weeds. Grass-weeds are over-represented in global cases of resistance. Some comment on this in discussion maybe. ***Done***

Discussion: The paper would benefit from a more concise discussion with improved, more logical structure. Content is OK but lacking in focus and a bit rambling. I suggest reducing it to about 60% max of current length by heavily reducing some paragraphs or omitting altogether. The focus should be on the lessons and implications from what you have presented in the paper. Some of Discussion, while valid, reduces the impact of the paper rather than enhances it.

226-235 Better if this is later in Discussion. ***Done***

236-261 Reduce this paragraph very substantially. Some of this could be in introduction.

There appear to be some contradictions quoted: '77 cases in wheat and 30 cases in barley [4].' ' A review of the New Zealand literature shows only two reports of

resistant species in wheat and barley [7,8].' 'Cases' is confusing as different, I think, to previous use of term. ***Done***

262-287 Suggest this starts the Discussion as seems more logical. ***Done***

288-306 Again lacks focus. Glyphosate comments relevant. But to make some suggestion but then say 'Estimations of these other factors can be made, but are likely to be inaccurate.' Is a case of 'shooting yourself in the foot.' Ditto 'Using Beckie's as our herbicide-risk ranking would produce different risk-scores.' Omit. ***Several sentences deleted and reworded to avoid "shooting ourselves in the foot" but keeping the main ideas as a reasonable caveat around interpretation of our results.***

Other paragraphs – consider what content really adds to paper and what can be omitted.

One aspect that seems missing, is some idea of the crop rotations used on NZ farms and the impact this might have. This focus in this paper is on wheat and barley but the frequency of growing these crops and consequent herbicide use will surely impact on the resistance risk? How commonly is arable cropping rotated with grass? Are all-arable farms common compared with mixed farms? This may explain why herbicide resistance is relatively uncommon in NZ. Also relevant to risk at individual field level which lends itself nicely to comments about where you should set priorities for resistance monitoring, management and farmer KT. No need to go into great detail, but is relevant. ***We have included some text about rotations.***

Conclusions: Generally fine but suggest the following.

343-346 Suggest: 'A European protocol [17], designed primarily to assist in herbicide authorization procedures, was adapted to assess the risk of herbicide resistance evolving in 100 different weed species known to occur in New Zealand wheat and barley crops. More than half the weeds (55) we assessed were low-risk, 29 were medium-risk and 16 high-risk. The 10 species posing the highest resistance risk were: etc etc' ***Done***

349-351 Final two sentences could usefully be a bit 'punchier'. 'Suggest: 'We are planning extensive surveys in New Zealand to detect new cases of herbicide resistance. The risk assessment outlined in this paper will enable us to prioritise those weeds identified as posing a high resistance risk and, consequently, make better use of available resources. The risk assessment procedure as described in this paper has the potential to be a very useful tool for evaluating the risk of herbicide resistance in a wide range of different weed species in other countries too'. ***Done***

Reviewer #3 (YASEEN KHALIL):

The introduction and methodology of the experiments seem to be satisfactory.

However, in the methods section, the authors need to explain how they did the statistical analyses and data presentation.

> ***We do not carry out any statistical tests. All our data is descriptive, and are visualized using graphs . We added a sentence to the last line of the methods section about the graphs.***

SPECIFIC COMMENTS:

L20-22 Add the classifiers to the scientific names. Alternatively, you may add the classifiers to the scientific names in the Materials and methods section and after that no need for it to be mentioned.

> ***We think the reviewer is referring to the specific binomial author authorities. We examined other abstracts in PLOS ONE and do not see the these being used for abstracts unless it is specifically about a taxonomic treatment.***

L20-22 Add hyphen "herbicide-resistant" ***Done but line 27***

L45 Add the "it reflects the varying" ***Done***

L52 Add the classifier to the scientific name "*Alopecurus myosuroides* ". ***Done and also for Avena fatua***

L20-22 Add hyphen "farm-scale" ***We added this at line 68 though which was the first mention***

L74-76 What about the other 25% of the wheat and barley production regions in New Zealand. I would prefer to stick to the mentioned region and not extrapolate to the whole country of New Zealand.

> ***We intend the weed list to capture our best estimate of the weeds known to occur in wheat and barley fields in New Zealand. We added a new sentence to explain: We expanded the weed species list to include species known to occur in wheat and barley fields in the wider New Zealand context.***

L78 It will be very useful to mention the regions of the studies conducted by the mentioned researcher's literature [21, 22].

We disagree. We checked the regions studied in those articles and knowing them adds little insight. Also, only a few species were added from these.

L79 It is recommended to follow the journal guidelines in this regards instead (Species nomenclature). ***Taxonomic authorities added at first mention.***

L91-92 What is the logic and the rationale behind this decision? Worldwide, Group A herbicides are the most vulnerable group in terms of weed resistance evolving

> ***We do classify Group A herbicides as high risk. We include a bit more detail here and consider the issues in the discussion. It now reads: With group A having 48 cases and group G herbicides having 47 cases we chose to place the two groups in the same risk category, with a difference in the numbers of cases of just worldwide we believe they are indistinguishable from the data. The alternative is to use the same threshold as in the Moss protocol, but this would result in group A and G being medium risk, which fails to capture the high-risk status of group A herbicides.***

L122 Add the classifier to the scientific name "*Chenopodium album*". ***Done***

L140 Add the "that the actual" ***Done***

L142 Add comma "distribution, and abundance" ***Done***

L147 Add of "total of 100 weeds" ***Done, but it is now 101 weeds.***

L150 I would recommend adding the S1 Table to the text instead of having it as a supporting file. ***Done***

L152 I would recommend adding the S2 Table to the text instead of having it as a supporting file. ***Done We think this one should stay where it is because of its length.***

L179 Add the "ranked by the wheat" ***Done***

L203 Add comma "modes-of-action, and" ***Done***

L234 Add comma "Without this list, we" ***Done***

L234 replace are with a "As a result" ***Done***

L240 Add comma "cases in wheat, and" ***Done***

L246 Add s "indicates elevated" ***Not done. "may indicate" is correct***

L247 Add comma "surprising when" ***Not done. Comma is correct***

L256 delete "Clearly" ***Done***

L275, 281, 284 Add hyphens to "spring-sown cereals" ***Done***, **"on-field"** ***Not done the acting on field experience" refers to experience gained in the field***, **"high-risk"** ***Done***

L289"because of the number" ***Not done. This makes sense "This was necessary in part because the number of cases for different herbicide groups has increased since the Moss article was published."***

L290 have instead of has "groups have increased" ***Not done. This makes sense "This was necessary in part because the number of cases for different herbicide groups has increased since the Moss article was published."***

L296“because of worldwide” ***Not done, it makes sense the way it is.***

L308 Surprisingly, there have ***Done***

L324 Add commas “may, in fact, implicate” ***Done***
