# Supplementary material for: A herbicide resistance risk assessment for weeds in wheat and barley crops in New Zealand
Source: PLoS One. 2020 Jun 25;15(6):e0234771. doi: 10.1371/journal.pone.0234771 (PMC7316288; doi:10.1371/journal.pone.0234771)
Supplement: Attachment — Submitted filename: Reviewers comments on PONE-D-20-09801 29April20.docx [file pone.0234771.s002.docx]

**PONE-D-20-09801**

**A herbicide resistance risk assessment for weeds in**

**wheat and barley crops in New Zealand**

**Ngow et al**

**Line No (as in downloaded pdf).**

**Abstract: Generally fine** but as likely to be most-read part I suggest a few minor changes. Do you need to say ‘wheat and barley’ or would ‘cereal’ suffice? Ditto in title.

I suggest:

15-18	‘We estimated the risk of selecting for herbicide resistance in 100 weed species known to occur in wheat and barley crops on farms in New Zealand. A protocol was used that accounts for both the risk that different herbicides will select for resistance and each weed’s propensity to develop herbicide resistance based on the number of cases worldwide.’

24	‘ALS-inhibitors were assessed as posing the greatest risk for more species than any	other mode-of-action.

25	I prefer ‘Pre-emergence’ but that may depend on journal style.

26	‘……. in this class commonly used by…..’ Is this better than ‘favoured’?

Somewhere I think it would be worth stressing in abstract that 7 out of the high-risk 10 are grass-weeds. If space is limiting, I think the last sentence of abstract could go or ‘farmer extension efforts’ incorporated into previous sentence.

**Introduction: Good – acceptably concise and relevant.** I suggest including some brief (one or two sentences) information on arable cropping in NZ and what proportion of that is accounted for by wheat and barley. Or at least to show that wheat and barley comprise a significant proportion of arable crops – you might even wish to mention that NZ currently holds world record for both barley and wheat yield/ha (I think that is correct). I would!

32	Suggest: ‘……potential losses….’ Losses of 23% don’t actually commonly occur.

41	Clarify ‘cases’. To non-specialist reader it is vague and could mean number of fields or farms – although ‘specialists’ know why that term is appropriate. I would suggest stating number of weed species instead which is 13 according to Heap website country info. That lists 19 ‘cases’ (29 April) although that duplicates some species. If it is 25 cases now (according to your reference), perhaps someone in NZ should provide Ian Heap with updated info. This is important if this paper is published. Likewise amend ‘12’ in line 42 if appropriate.

45	‘haphazard’ is a bit unfair, although this is a valid point. I would dispute that my sampling and testing over 30 years has been ‘haphazard’! Suggest ‘unsystematic’ as a better word – I would agree with that. End sentence after ‘globally’. Then ‘It reflects…..’

50	comma needed after [9-14]. These 6 refs are all valid, but do you need them all?

53-54	Good points – one issue is that if resistance is perceived to be rare then hard to justify cost. If very widespread then why bother to do a survey? Money could be better spent. Also lack of infrastructure and personnel to conduct surveys may be as important as cost. Also, do surveys have much long-term value? Is a 20-year old survey of much value now – perhaps only as a benchmark? Perhaps money better spent on more ‘durable’ studies? Not saying that you need to consider these aspects here – perhaps in discussion as risk assessment relevant to survey priorities.

59	Suggest: ‘…….and their prior record of resistance in cereal fields elsewhere in the world.’

62-64	This is fine and a good succinct sentence, but I wonder if it is worth emphasising more that risk assessment includes not only herbicide risk but also weed species risk for non-specialist readers, as this is not particularly intuitive? So, could read: ‘They present a quantitative risk matrix using both herbicide-risk (some herbicides pose a higher risk than others) and species-risk (some weed species are more resistance-prone than others), with an optional score modifier designed to account for agronomic management practices that may reduce the risk.’

67	Change: ‘Individual field and farm scale risk is not assessed as this requires detailed information on past herbicide use, including timing etc…..’ (This covers what has been applied - surely most important factor?)

**Materials and methods: Good – makes it clear what was done and why slightly different approaches to published protocol were adopted**

76	Suggest: ‘Most grasses and some …..’ The wonders of Google mean that I can see, within 60 seconds, that two grass weed species were identified at species level in the paper cited…..

83	Suggest: ‘….legacy herbicide mode of action (MoA) groups……’

88-89	Suggest: ‘….legacy HRAC MoA group [24], with risk scores of 1, 2 or 3 given for low, medium or high risk respectively.’

96	Suggest: ‘…..scored as ‘1’.’ (‘One’ is a bit ambiguous).

98	Suggest: ‘Most recent….’

108-109	This is slightly confusing: ‘The most used herbicides for each crop were characterized by weighting active ingredient amounts and the number of fields they were applied to.’ It almost implies total a.i. weight was used. Not sure this sentence is needed.

110	Why taxon? Species better?

112	Suggest: ‘…..HRAC MoA group…...’ And I suggest elsewhere throughout paper.

113	Suggest: ‘….herbicide type….

114	Suggest: ‘……the global number of resistance cases….’

115	Suggest: ‘To obtain the ‘high’, ‘medium’ and ‘low’ risk scores as used in the	Moss protocol [17],…..’

117-118	This sentence is slightly confusing and maybe could be improved. Is this correct? ‘We assessed overall species-risk as the sum of the herbicide-risk multiplied by the “inherent” species-risk [17] combined for all relevant HRAC MoA herbicide groups, but only…….’ (I think ‘once’ is confusing). The example you give below is useful.

120	Suggest: ’……weed species….’

135	Suggest: ‘……to determine species-risk scores based on the number of cases of resistance worldwide, but we think this……’

137	Suggest: ‘ ….45 ‘high’ and ‘medium’ resistance risk species, many more than Moss et al……….’

139	Suggest: ‘The Moss protocol also used score modifiers that take into account resistance management practices including the use of non-chemical control measures’ .

139-142	Suggest: ‘We did not use the score modifiers since these vary from field to field and our objective was slightly different. We acknowledge that actual risk of resistance development is determined mostly by the frequency and type of herbicide applied (selection pressure) interacting with characteristics of weed biology, distribution and abundance [5].’

**Results: Generally good and concise. One key paragraph needs improvement to improve clarity**

145	Suggest: ‘An additional 31 species were added…..’

147	Suggest: ‘ ….resulting in a total of 100 weed species for consideration.’

155	Suggest: Suggest: ‘This resembles the table of Moss et al., [17] with…’

Table 1	Use of ‘taxa’ is odd. Why not ‘species’ which is what is used on Heap’s website? Taxa is a rather more general term and I cannot see any justification for use here. Would it also be useful to make the herbicide example the most commonly used ai for the group in NZ? Or if this has been done, state in title.

171-172	Clarify if glyphosate used in-crop (for desiccation) or pre-sowing or both. Presumably both, but some comment about balance of use would be useful if only to stress that glyphosate use is very different to selective herbicides – non-specialists might assume use is primarily in GM crops, as in some other countries. Either here or earlier in M&M.

Suggest: ‘ …….in barley (18%) compared with wheat (12%); conversely, farmers………’

174	Again, ‘HRAC MoA categories’

Table 2	Suggest last 10 categories are simply summarised to save space and some statement added within table. e.g. ‘The following 10 HRAC MoA groups each accounted for less than 1% of herbicide applications, N, F4, K1, I, H, F3, Z, L, F2 & K2.’

181	I don’t really see the point of stating actual % for barley when you haven’t for wheat. Why not simply add % values to each of the bars in Fig 1. You could then say a bit about relative use of some individual herbicides in wheat and barley. Somewhere you should give some indication of relative area sown with wheat and barley – in introduction or M&M.

183	Suggest: ‘Flufenacet and terbuthylazine were the only herbicides used on a significant area pre-emergence (the latter can also be used post-emergence), both used less in barley crops.’

185	Suggest: ‘**Fig 1. The ten most commonly applied herbicides in New Zealand wheat and barley fields**.’

190	Why taxon? Surely ‘species’ is better?

193-194	Say eight grasses and eight broad-leafed weeds.

201	Suggest: ‘….five or more unique mode-of-action groups each’ What does>20 mean? Clarify.

202-215	This paragraph covers the key output of this paper but is confusing and really needs a thorough re-write. Expand if necessary, to clarify results. Fig 2 contains a lot of information. I did wonder how much the ‘cases’ adds to this but I can see that it is relevant. Some definition of ‘cases’ is needed, as mentioned earlier. This is tricky as not easy to explain in a few words, but if this is explained in the M&M then no need to explain again, except perhaps to say refer to M&M. Should be made clear in Figure that cases is worldwide, as might be interpreted by casual reader as in NZ.

212-215	I would make the point that 7 out of 10 are grass-weeds. Grass-weeds are over-represented in global cases of resistance. Some comment on this in discussion maybe.

**Discussion: The paper would benefit from a more concise discussion with improved, more logical structure. Content is OK but lacking in focus and a bit rambling. I suggest reducing it to about 60% max of current length by heavily reducing some paragraphs or omitting altogether. The focus should be on the lessons and implications from what you have presented in the paper. Some of Discussion, while valid, reduces the impact of the paper rather than enhances it.**

226-235	Better if this is later in Discussion.

236-261	Reduce this paragraph very substantially. Some of this could be in introduction.

There appear to be some contradictions quoted: ‘77 cases in wheat and 30 cases in barley [4].’ ‘ A review of the New Zealand literature shows only two reports of resistant species in wheat and barley [7,8].’ ‘Cases’ is confusing as different, I think, to previous use of term.

262-287	Suggest this starts the Discussion as seems more logical.

288-306	Again lacks focus. Glyphosate comments relevant. But to make some suggestion but then say ‘Estimations of these other factors can be made, but are likely to be inaccurate.’ Is a case of ‘shooting yourself in the foot.’ Ditto ‘Using Beckie’s as our herbicide-risk ranking would produce different risk-scores.’ Omit.

Other paragraphs – consider what content really adds to paper and what can be omitted.

One aspect that seems missing, is some idea of the crop rotations used on NZ farms and the impact this might have. This focus in this paper is on wheat and barley but the frequency of growing these crops and consequent herbicide use will surely impact on the resistance risk? How commonly is arable cropping rotated with grass? Are all-arable farms common compared with mixed farms? This may explain why herbicide resistance is relatively uncommon in NZ. Also relevant to risk at individual field level which lends itself nicely to comments about where you should set priorities for resistance monitoring, management and farmer KT. No need to go into great detail, but is relevant.

**Conclusions: Generally fine but suggest the following.**

343-346	Suggest: **‘**A European protocol [17], designed primarily to assist in herbicide authorization procedures, was adapted to assess the risk of herbicide resistance evolving in 100 different weed species known to occur in New Zealand wheat and barley crops. More than half the weeds (55) we assessed were low-risk, 29 were medium-risk and 16 high-risk. The 10 species posing the highest resistance risk were: etc etc’

349-351	Final two sentences could usefully be a bit ‘punchier’. ’Suggest: ‘We are planning extensive surveys in New Zealand to detect new cases of herbicide resistance. The risk assessment outlined in this paper will enable us to prioritise those weeds identified as posing a high resistance risk and, consequently, make better use of available resources. The risk assessment procedure as described in this paper has the potential to be a very useful tool for evaluating the risk of herbicide resistance in a wide range of different weed species in other countries too’.
