# Supplementary material for: A herbicide resistance risk assessment for weeds in wheat and barley crops in New Zealand
Source: PLoS One. 2020 Jun 25;15(6):e0234771. doi: 10.1371/journal.pone.0234771 (PMC7316288; doi:10.1371/journal.pone.0234771)
Supplement: Attachment — Submitted filename: PONE-D-20-09801_reviewer (YK).docx [file pone.0234771.s003.docx]

**Reviewer (YASEEN KHALIL):**

Thanks for allowing me to review the manuscript entitle “A herbicide resistance risk assessment for weeds in wheat and barley crops in New Zealand”. This is a very well written and informative manuscript. Authors have carried out a systematic study to determine the quantitative risk matrix using ranked herbicide-risk and species-risk with optional score modifier designed to account for agronomic management practices that may reduce the risk. The researchers took advantage of a unique data set about herbicide use in wheat and barley fields in New Zealand to place their risk assessment into context, and construct a framework for herbicide resistance surveys and extension efforts in the New Zealand cropping industry. This study would be provided a very good message for the growers about the herbicide resistance risk assessment on the industry-wide scale, but not on the field and farm scale. I am in favor of publishing this manuscript to the PLOS ONE Journal. I have very few comments.

**GENERAL COMMENTS:**

The manuscript represents a review article instead of research article, as it is been mentioned in the manuscript draft, in an important area of herbicide research and was conducted with reasonable methods. In addition, the manuscript is written well, with areas for improvement in several instances.

Keywords should be main words that are not included in the title and the abstract.

The abstract is well structured. I suggest mentioning the scientific name of the weeds including the name of classifiers for the first time.

The introduction and methodology of the experiments seem to be satisfactory. However, in the methods section, the authors need to explain how they did the statistical analyses and data presentation.

**SPECIFIC COMMENTS:**

L20-22 Add the classifiers to the scientific names. Alternatively, you may add the classifiers to the scientific names in the Materials and methods section and after that no need for it to be mentioned.

L20-22 Add hyphen “herbicide-resistant”

L45 Add the “it reflects the varying”

L52 Add the classifier to the scientific name “*Alopecurus myosuroides* ”.

L20-22 Add hyphen “farm-scale”

L74-76 What about the other 25% of the wheat and barley production regions in New Zealand. I would prefer to stick to the mentioned region and not extrapolate to the whole country of New Zealand.

L78 It will be very useful to mention the regions of the studies conducted by the mentioned researcher’s literature [21, 22].

L79 It is recommended to follow the journal guidelines in this regards instead (Species nomenclature).

L91-92 What is the logic and the rationale behind this decision? Worldwide, Group A herbicides are the most vulnerable group in terms of weed resistance evolving

L122 Add the classifier to the scientific name “*Chenopodium album*”.

L140 Add the “that the actual”

L142 Add comma “distribution, and abundance”

L147 Add of “total of 100 weeds”

L150 I would recommend adding the S1 Table to the text instead of having it as a supporting file.

L152 I would recommend adding the S2 Table to the text instead of having it as a supporting file.

L179 Add the “ranked by the wheat”

L203 Add comma “modes-of-action, and”

L234 Add comma “Without this list, we”

L234 replace are with a “As a result”

L240 Add comma “cases in wheat, and”

L246 Add s “indicates elevated”

L247 Add comma “surprising when”

L256 delete “Clearly”

L275, 281, 284 Add hyphens to “spring-sown cereals”, “on-field”, “high-risk”

L289“because of the number”

L290 have instead of has “groups have increased”

L296“because of worldwide”

L308 Surprisingly, there have

L324 Add commas “may, in fact, implicate”
