# Supplementary material for: A herbicide resistance risk assessment for weeds in wheat and barley crops in New Zealand
Source: PLoS One. 2020 Jun 25;15(6):e0234771. doi: 10.1371/journal.pone.0234771 (PMC7316288; doi:10.1371/journal.pone.0234771)
Supplement: Attachment — Submitted filename: Response to Reviewers.docx [file pone.0234771.s004.docx]

## Reviewer #1:

Because frequency and abundance of weed species in the region have not been used in the scoring, the paper provides a picture of risks due to the current use of herbicides in the region, which is informative for the farmer about the intrinsic risk of an abundant species in its own farm. However, although I agree with intrinsic high risk species as stated on line 280-285, it is pity that the overall risk for the region is not presented.

***The regional risk is assessed! We use a published account of weeds, as well as expert knowledge to identify problem weeds in wheat and barley. The risk assessment considers the propensity of those weeds to develop resistance. It uses the most important factors, the crop specific region wide weed flora and region wide data about herbicide use. The selection pressure comes from the herbicides used and so captures the most important dimensions of the regional risk.***

**L50-54** There is also an important survey on blackgrass in UK (see Hicks et al Nature Ecology & Evolution 2(3) 2018, DOI: 10.1038/s41559-018-0470-1). Probably other reports are available on surveys developed by national weed research associations in Spain, France (Columa), Italy, etc., including companies, extension service and farmers, and published in local conferences.

***That is another example of the “focus on one or two problematic species in a given crop” issue we highlight, and therefore does not need to be added.***

**L142** It can be assumed that weed biology is already included in the inherent species score, but abundance is a key parameter. Abundance includes effects of farming systems: if many individuals still remain in the field, then the risk of developing resistance is higher than if the population is small. A score modifier to account for variation of abundance (e.g. 1 for low density and 3 for more than a million plant/ha, or another scale) could be used. Another factor is the species frequency in the studied region, which doesn’t seem to be taken into account (is the Production Wise platform collecting main troublesome weed species?).

***While that is true, regional abundance and density data do not exist to be able to implement that. We acknowledge that distribution, abundance and phenology are important #(L272). ProductionWise does not gather any information on weeds. Because we identified high-risk species and herbicides the data are useful as a regional assessment. Farmers and experts can now interpret the observed weed abundance with an eye to the likely risk of resistance.***

**Fig 2** Rather the low and high use HRAC groups I expected to see also the number of cases in New Zealand. I’m not clear with the low versus high use HRAC groups.

***We emphasize high use herbicides in our studied regions, because that selection pressure drives the evolution of resistance. We mention in the introduction that in New Zealand, there are only 12 instances of herbicide resistance in arable crops #(L41-43) and in the discussion delve further into New Zealand HR weeds (L238-244). The high and low usage herbicide groups come from ProductionWise data, which can be found on Table 2. The basis of deciding which groups are ‘high use in New Zealand’ is on L106-108. Cases of resistance have been indicated on Table 3.***

## Reviewer #2 Dr Stephen Moss:

**Abstract: Generally fine** but as likely to be most-read part I suggest a few minor changes. Do you need to say ‘wheat and barley’ or would ‘cereal’ suffice? Ditto in title.

***We think wheat and barley is a more accurate statement for our case since the herbicide data focuses on that. Wheat and barley is more widely grown than oats, and depending on the reader, corn would also be considered to be a cereal…***

I suggest:

**15-18**	‘We estimated the risk of selecting for herbicide resistance in 100 weed species known to occur in wheat and barley crops on farms in New Zealand. A protocol was used that accounts for both the risk that different herbicides will select for resistance and each weed’s propensity to develop herbicide resistance based on the number of cases worldwide.’ ***Done***

24	‘ALS-inhibitors were assessed as posing the greatest risk for more species than any	other mode-of-action. ***Done***

25	I prefer ‘Pre-emergence’ but that may depend on journal style. ***Done***

26	‘……. in this class commonly used by…..’ Is this better than ‘favoured’? ***Done***

Somewhere I think it would be worth stressing in abstract that 7 out of the high-risk 10 are grass-weeds. If space is limiting, I think the last sentence of abstract could go or ‘farmer extension efforts’ incorporated into previous sentence. ***Done***

**Introduction: Good – acceptably concise and relevant.** I suggest including some brief (one or two sentences) information on arable cropping in NZ and what proportion of that is accounted for by wheat and barley. Or at least to show that wheat and barley comprise a significant proportion of arable crops – you might even wish to mention that NZ currently holds world record for both barley and wheat yield/ha (I think that is correct). I would! ***Done***

32	Suggest: ‘……potential losses….’ Losses of 23% don’t actually commonly occur. ***Done***

41	Clarify ‘cases’. To non-specialist reader it is vague and could mean number of fields or farms – although ‘specialists’ know why that term is appropriate. I would suggest stating number of weed species instead which is 13 according to Heap website country info. That lists 19 ‘cases’ (29 April) although that duplicates some species. If it is 25 cases now (according to your reference), perhaps someone in NZ should provide Ian Heap with updated info. This is important if this paper is published. Likewise amend ‘12’ in line 42 if appropriate. ***Done reworded to reflect species numbers in New Zealand, and documented instances of resistance, to distinguish from the usage of cases in the Heap database.***

45	‘haphazard’ is a bit unfair, although this is a valid point. I would dispute that my sampling and testing over 30 years has been ‘haphazard’! Suggest ‘unsystematic’ as a better word – I would agree with that. End sentence after ‘globally’. Then ‘It reflects…..’ ***Done***

50	comma needed after [9-14]. These 6 refs are all valid, but do you need them all? ***They are valid, focus on systematic efforts to detect any and all weed resistant cases. Also, they could be hard to find for other researchers working on a similar project. Leaving as is.***

53-54	Good points – one issue is that if resistance is perceived to be rare then hard to justify cost. If very widespread then why bother to do a survey? Money could be better spent. Also lack of infrastructure and personnel to conduct surveys may be as important as cost. Also, do surveys have much long-term value? Is a 20-year old survey of much value now – perhaps only as a benchmark? Perhaps money better spent on more ‘durable’ studies? Not saying that you need to consider these aspects here – perhaps in discussion as risk assessment relevant to survey priorities. ***Good points thanks for the insightful commentary.***

59	Suggest: ‘…….and their prior record of resistance in cereal fields elsewhere in the world.’ ***We specifically looked at wheat and barley. It’s an important distinction.***

62-64	This is fine and a good succinct sentence, but I wonder if it is worth emphasising more that risk assessment includes not only herbicide risk but also weed species risk for non-specialist readers, as this is not particularly intuitive? So, could read: ‘They present a quantitative risk matrix using both herbicide-risk (some herbicides pose a higher risk than others) and species-risk (some weed species are more resistance-prone than others), with an optional score modifier designed to account for agronomic management practices that may reduce the risk.’ ***Done***

67	Change: ‘Individual field and farm scale risk is not assessed as this requires detailed information on past herbicide use, including timing etc…..’ (This covers what has been applied - surely most important factor?) ***We changed the paragraph to emphasize importance of our herbicide application data set: “. This risk assessment is on an industry-wide scale informed by anonymized herbicide application data from wheat and barley fields. Risks were not assessed at the scale of individual farms and fields, this requires detailed information about herbicide timing, mixtures and rotations, and their interactions with weed biology, crop rotations and other cultural practices. All the high-risk weeds identified here should be targeted in surveys designed to detect herbicide-resistant weeds.***

**Materials and methods: Good – makes it clear what was done and why slightly different approaches to published protocol were adopted**

76	Suggest: ‘Most grasses and some …..’ The wonders of Google mean that I can see, within 60 seconds, that two grass weed species were identified at species level in the paper cited….. ***Done***

83	Suggest: ‘….legacy herbicide mode of action (MoA) groups……’ ***Done***

88-89	Suggest: ‘….legacy HRAC MoA group [24], with risk scores of 1, 2 or 3 given for low, medium or high risk respectively.’ ***Done***

96	Suggest: ‘…..scored as ‘1’.’ (‘One’ is a bit ambiguous). ***Done***

98	Suggest: ‘Most recent….’ ***Done but, “The most recent…”***

108-109	This is slightly confusing: ‘The most used herbicides for each crop were characterized by weighting active ingredient amounts and the number of fields they were applied to.’ It almost implies total a.i. weight was used. Not sure this sentence is needed. **Deleted the sentence.**

110	Why taxon? Species better? ***Changed to species.***

112	Suggest: ‘…..HRAC MoA group…...’ And I suggest elsewhere throughout paper. ***Done***

113	Suggest: ‘….herbicide type…. ***Done***

114	Suggest: ‘……the global number of resistance cases….’	***Done***

115	Suggest: ‘To obtain the ‘high’, ‘medium’ and ‘low’ risk scores as used in the	Moss protocol [17],…..’ ***Done***

117-118	This sentence is slightly confusing and maybe could be improved. Is this correct? ‘We assessed overall species-risk as the sum of the herbicide-risk multiplied by the “inherent” species-risk [17] combined for all relevant HRAC MoA herbicide groups, but only…….’ (I think ‘once’ is confusing). The example you give below is useful. ***Done***

120	Suggest: ’……weed species….’ ***Done***

135	Suggest: ‘……to determine species-risk scores based on the number of cases of resistance worldwide, but we think this……’ ***Done***

137	Suggest: ‘ ….45 ‘high’ and ‘medium’ resistance risk species, many more than Moss et al……….’ ***Done***

139	Suggest: ‘The Moss protocol also used score modifiers that take into account resistance management practices including the use of non-chemical control measures’ . ***Done***

139-142	Suggest: ‘We did not use the score modifiers since these vary from field to field and our objective was slightly different. We acknowledge that actual risk of resistance development is determined mostly by the frequency and type of herbicide applied (selection pressure) interacting with characteristics of weed biology, distribution and abundance [5].’ ***Done***

**Results: Generally good and concise. One key paragraph needs improvement to improve clarity**

145	Suggest: ‘An additional 31 species were added…..’ ***Done***

147	Suggest: ‘ ….resulting in a total of 100 weed species for consideration.’ ***Done***

155	Suggest: Suggest: ‘This resembles the table of Moss et al., [17] with…’ ***Done***

Table 1	Use of ‘taxa’ is odd. Why not ‘species’ which is what is used on Heap’s website? Taxa is a rather more general term and I cannot see any justification for use here. Would it also be useful to make the herbicide example the most commonly used ai for the group in NZ? Or if this has been done, state in title. ***Done***

171-172	Clarify if glyphosate used in-crop (for desiccation) or pre-sowing or both. Presumably both, but some comment about balance of use would be useful if only to stress that glyphosate use is very different to selective herbicides – non-specialists might assume use is primarily in GM crops, as in some other countries. Either here or earlier in M&M. ***Done “Glyphosate (MoA group G) is mostly used (>95%) used pre-sowing of the cereal crops, for termination of the previous crop or pre-establishment weed control. It is very rarely used as crop pre-harvest desiccant.”***

Suggest: ‘ …….in barley (18%) compared with wheat (12%); conversely, farmers………’ ***Done***

174	Again, ‘HRAC MoA categories’ ***Done***

Table 2	Suggest last 10 categories are simply summarised to save space and some statement added within table. e.g. ‘The following 10 HRAC MoA groups each accounted for less than 1% of herbicide applications, N, F4, K1, I, H, F3, Z, L, F2 & K2.’ ***Done***

181	I don’t really see the point of stating actual % for barley when you haven’t for wheat. Why not simply add % values to each of the bars in Fig 1. You could then say a bit about relative use of some individual herbicides in wheat and barley. Somewhere you should give some indication of relative area sown with wheat and barley – in introduction or M&M. ***We added the percent values to the graph and clarified a comment about relative use under Table 2. The areas sown for wheat and barley are now in the introduction.***

183	Suggest: ‘Flufenacet and terbuthylazine were the only herbicides used on a significant area pre-emergence (the latter can also be used post-emergence), both used less in barley crops.’

185	Suggest: ‘**Fig 1. The ten most commonly applied herbicides in New Zealand wheat and barley fields**.’ ***Done***

190	Why taxon? Surely ‘species’ is better? ***Done***

193-194	Say eight grasses and eight broad-leafed weeds. ***Done***

201	Suggest: ‘….five or more unique mode-of-action groups each’ What does>20 mean? Clarify. ***Done***

202-215	This paragraph covers the key output of this paper but is confusing and really needs a thorough re-write. Expand if necessary, to clarify results. Fig 2 contains a lot of information. I did wonder how much the ‘cases’ adds to this but I can see that it is relevant. Some definition of ‘cases’ is needed, as mentioned earlier. This is tricky as not easy to explain in a few words, but if this is explained in the M&M then no need to explain again, except perhaps to say refer to M&M. Should be made clear in Figure that cases is worldwide, as might be interpreted by casual reader as in NZ. ***Done*** ***Figure 2 clearly mentions global cases. Also added the following to the methods: “Cases are defined by the International Survey of Herbicide Resistant Weeds as unique combinations of weed species and HRAC herbicide mode-of-action (species x site of action).”***

212-215	I would make the point that 7 out of 10 are grass-weeds. Grass-weeds are over-represented in global cases of resistance. Some comment on this in discussion maybe. ***Done***

**Discussion: The paper would benefit from a more concise discussion with improved, more logical structure. Content is OK but lacking in focus and a bit rambling. I suggest reducing it to about 60% max of current length by heavily reducing some paragraphs or omitting altogether. The focus should be on the lessons and implications from what you have presented in the paper. Some of Discussion, while valid, reduces the impact of the paper rather than enhances it.**

226-235	Better if this is later in Discussion. ***Done***

236-261	Reduce this paragraph very substantially. Some of this could be in introduction.

There appear to be some contradictions quoted: ‘77 cases in wheat and 30 cases in barley [4].’ ‘ A review of the New Zealand literature shows only two reports of resistant species in wheat and barley [7,8].’ ‘Cases’ is confusing as different, I think, to previous use of term. ***Done***

262-287	Suggest this starts the Discussion as seems more logical. ***Done***

288-306	Again lacks focus. Glyphosate comments relevant. But to make some suggestion but then say ‘Estimations of these other factors can be made, but are likely to be inaccurate.’ Is a case of ‘shooting yourself in the foot.’ Ditto ‘Using Beckie’s as our herbicide-risk ranking would produce different risk-scores.’ Omit. ***Several sentences deleted and reworded to avoid “shooting ourselves in the foot” but keeping the main ideas as a reasonable caveat around interpretation of our results.***

Other paragraphs – consider what content really adds to paper and what can be omitted.

One aspect that seems missing, is some idea of the crop rotations used on NZ farms and the impact this might have. This focus in this paper is on wheat and barley but the frequency of growing these crops and consequent herbicide use will surely impact on the resistance risk? How commonly is arable cropping rotated with grass? Are all-arable farms common compared with mixed farms? This may explain why herbicide resistance is relatively uncommon in NZ. Also relevant to risk at individual field level which lends itself nicely to comments about where you should set priorities for resistance monitoring, management and farmer KT. No need to go into great detail, but is relevant. ***We have included some text about rotations.***

**Conclusions: Generally fine but suggest the following.**

343-346	Suggest: **‘**A European protocol [17], designed primarily to assist in herbicide authorization procedures, was adapted to assess the risk of herbicide resistance evolving in 100 different weed species known to occur in New Zealand wheat and barley crops. More than half the weeds (55) we assessed were low-risk, 29 were medium-risk and 16 high-risk. The 10 species posing the highest resistance risk were: etc etc’ ***Done***

349-351	Final two sentences could usefully be a bit ‘punchier’. ’Suggest: ‘We are planning extensive surveys in New Zealand to detect new cases of herbicide resistance. The risk assessment outlined in this paper will enable us to prioritise those weeds identified as posing a high resistance risk and, consequently, make better use of available resources. The risk assessment procedure as described in this paper has the potential to be a very useful tool for evaluating the risk of herbicide resistance in a wide range of different weed species in other countries too’. ***Done***

## Reviewer #3 (YASEEN KHALIL):

The introduction and methodology of the experiments seem to be satisfactory.

However, in the methods section, the authors need to explain how they did the statistical analyses and data presentation.

***We do not carry out any statistical tests. All our data is descriptive, and are visualized using graphs . We added a sentence to the last line of the methods section about the graphs.***

**SPECIFIC COMMENTS:**

**L20**-22 Add the classifiers to the scientific names. Alternatively, you may add the classifiers to the scientific names in the Materials and methods section and after that no need for it to be mentioned.

***We think the reviewer is referring to the specific binomial author authorities. We examined other abstracts in PLOS ONE and do not see the these being used for abstracts unless it is specifically about a taxonomic treatment.***

**L20**-22 Add hyphen “herbicide-resistant” ***Done but line 27***

**L45** Add the “it reflects the varying” ***Done***

**L52** Add the classifier to the scientific name “*Alopecurus myosuroides* ”. ***Done and also for Avena fatua***

**L20**-22 Add hyphen “farm-scale” ***We added this at line 68 though which was the first mention***

**L74**-76 What about the other 25% of the wheat and barley production regions in New Zealand. I would prefer to stick to the mentioned region and not extrapolate to the whole country of New Zealand.

***We intend the weed list to capture our best estimate of the weeds known to occur in wheat and barley fields in New Zealand. We added a new sentence to explain: We expanded the weed species list to include species known to occur in wheat and barley fields in the wider New Zealand context.***

**L78** It will be very useful to mention the regions of the studies conducted by the mentioned researcher’s literature [21, 22].

***We disagree. We checked the regions studied in those articles and knowing them adds little insight. Also, only a few species were added from these.***

**L79** It is recommended to follow the journal guidelines in this regards instead (Species nomenclature). ***Taxonomic authorities added at first mention.***

**L91-92** What is the logic and the rationale behind this decision? Worldwide, Group A herbicides are the most vulnerable group in terms of weed resistance evolving

***We do classify Group A herbicides as high risk. We include a bit more detail here and consider the issues in the discussion. It now reads: With group A having 48 cases and group G herbicides having 47 cases we chose to place the two groups in the same risk category, with a difference in the numbers of cases of just worldwide we believe they are indistinguishable from the data. The alternative is to use the same threshold as in the Moss protocol, but this would result in group A and G being medium risk, which fails to capture the high-risk status of group A herbicides.***

**L122** Add the classifier to the scientific name “*Chenopodium album*”. ***Done***

**L140** Add the “that the actual” ***Done***

**L142** Add comma “distribution, and abundance” ***Done***

**L147** Add of “total of 100 weeds” ***Done, but it is now 101 weeds.***

**L150** I would recommend adding the S1 Table to the text instead of having it as a supporting file. ***Done***

**L152** I would recommend adding the S2 Table to the text instead of having it as a supporting file. ***Done* *We think this one should stay where it is because of its length.***

**L179** Add the “ranked by the wheat” ***Done***

**L203** Add comma “modes-of-action, and” ***Done***

**L234** Add comma “Without this list, we” ***Done***

**L234** replace are with a “As a result” ***Done***

**L240** Add comma “cases in wheat, and” ***Done***

**L246** Add s “indicates elevated” ***Not done. “may indicate” is correct***

**L247** Add comma “surprising when” ***Not done. Comma is correct***

**L256** delete “Clearly” ***Done***

**L275, 281, 284** Add hyphens to “spring-sown cereals” ***Done*, “on-field” *Not done the acting on field experience” refers to experience gained in the field*, “high-risk” *Done***

**L289**“because of the number” ***Not done. This makes sense “This was necessary in part because the number of cases for different herbicide groups has increased since the Moss article was published.”***

**L290** have instead of has “groups have increased” ***Not done. This makes sense “This was necessary in part because the number of cases for different herbicide groups has increased since the Moss article was published.”***

**L296**“because of worldwide” ***Not done, it makes sense the way it is.***

**L308** Surprisingly, there have ***Done***

**L324** Add commas “may, in fact, implicate” ***Done***
